# Bromodomain Proteins Contribute to Maintenance of Bloodstream Form Stage Identity in the African Trypanosome

**DOI:** 10.1371/journal.pbio.1002316

**Published:** 2015-12-08

**Authors:** Danae Schulz, Monica R. Mugnier, Eda-Margaret Paulsen, Hee-Sook Kim, Chun-wa W. Chung, David F. Tough, Inmaculada Rioja, Rab K. Prinjha, F. Nina Papavasiliou, Erik W. Debler

**Affiliations:** 1 Laboratory of Lymphocyte Biology, The Rockefeller University, New York, New York, United States of America; 2 Laboratory of Cell Biology, The Rockefeller University, New York, New York, United States of America; 3 Computational and Structural Chemistry, GlaxoSmithKline R&D, Medicines Research Centre, Stevenage, United Kingdom; 4 Epinova DPU, Immuno-Inflammation Therapy Area, GlaxoSmithKline, Medicines Research Centre, Stevenage, United Kingdom; University of Michigan, UNITED STATES

## Abstract

*Trypanosoma brucei*, the causative agent of African sleeping sickness, is transmitted to its mammalian host by the tsetse. In the fly, the parasite’s surface is covered with invariant procyclin, while in the mammal it resides extracellularly in its bloodstream form (BF) and is densely covered with highly immunogenic Variant Surface Glycoprotein (VSG). In the BF, the parasite varies this highly immunogenic surface VSG using a repertoire of ~2500 distinct *VSG* genes. Recent reports in mammalian systems point to a role for histone acetyl-lysine recognizing bromodomain proteins in the maintenance of stem cell fate, leading us to hypothesize that bromodomain proteins may maintain the BF cell fate in trypanosomes. Using small-molecule inhibitors and genetic mutants for individual bromodomain proteins, we performed RNA-seq experiments that revealed changes in the transcriptome similar to those seen in cells differentiating from the BF to the insect stage. This was recapitulated at the protein level by the appearance of insect-stage proteins on the cell surface. Furthermore, bromodomain inhibition disrupts two major BF-specific immune evasion mechanisms that trypanosomes harness to evade mammalian host antibody responses. First, monoallelic expression of the antigenically varied VSG is disrupted. Second, rapid internalization of antibodies bound to VSG on the surface of the trypanosome is blocked. Thus, our studies reveal a role for trypanosome bromodomain proteins in maintaining bloodstream stage identity and immune evasion. Importantly, bromodomain inhibition leads to a decrease in virulence in a mouse model of infection, establishing these proteins as potential therapeutic drug targets for trypanosomiasis. Our 1.25Å resolution crystal structure of a trypanosome bromodomain in complex with I-BET151 reveals a novel binding mode of the inhibitor, which serves as a promising starting point for rational drug design.

## Abstract 2

The use of specific inhibitors reveals a role for bromodomain proteins in epigenetically switching from the tsetse fly form of the sleeping sickness parasite *Trypanosoma brucei* to the mammalian bloodstream form.

## Author Summary

Many parasites undergo major changes in lifestyle as they cycle between their vectors and their hosts. We use *Trypanosoma brucei*, the causative agent of human and animal African Trypanosomiasis, as a model to study how these changes are regulated and maintained at the level of gene expression. *T*. *brucei* lives in the bloodstream of the mammalian host before migrating to the insect through the bite of its insect vector, the tsetse fly. It escapes immune detection by varying the proteins on its surface and by rapidly internalizing host antibodies. We report here that a class of proteins, called bromodomain proteins, help maintain the identity of the parasite in its bloodstream form. When these proteins are inhibited, the parasites express an unvarying epitope that is usually expressed only at the insect stage and are compromised in their ability to internalize host antibodies. Bromodomain proteins bind to DNA that is wrapped around histone proteins, acting as mediators that interact with the transcription machinery to determine which genes are turned on and which are kept repressed. Our crystal structure of a bromodomain inhibitor in complex with a trypanosome bromodomain reveals a novel binding mode and demonstrates how these small molecule inhibitors could be optimized for therapeutic use in a parasite-specific manner.

## Introduction

*Trypanosoma brucei* is a unicellular, protozoan parasite and the causative agent of Human African Trypanosomiasis (sleeping sickness). It also causes n'agana in cattle, a disease that imposes a severe economic burden in affected areas. The life cycle of *Trypanosoma brucei* requires adaptation to two distinct habitats: the fly (tsetse) and the bloodstream of its mammalian hosts. Within these habitats, the parasite assumes a succession of proliferative and quiescent developmental forms, which vary widely in metabolism, motility, and composition of the surface coat that covers the plasma membrane. In the fly, the trypanosome first resides in the midgut in its procyclic form (PF), where its surface is coated with a group of proteins collectively termed procyclins, and then in the salivary glands in its metacyclic form, where surface procyclin is replaced with a dense coat of Variant Surface Glycoprotein (VSG). The bite of the tsetse transmits the parasite to the mammalian host, where it resides extracellularly in its bloodstream form (BF) and continues to express VSG. The parasite relies on two strategies to evade the mammalian host antibody response. First, it varies (“switches”) its highly immunogenic surface antigen, using a repertoire of ~2,500 distinct *VSG* genes [1]. Only one *VSG* is expressed at a time (monoallelic expression), and host antibodies mounted against the initially expressed VSG must be continually replaced by antibodies against antigenically distinct VSGs, resulting in waves of parasitemia in the infected host [2,3]. Second, antibodies bound to surface VSG are rapidly internalized by the parasite [4], giving host effector cells less time to recognize and eliminate it. When trypanosomes enter the midgut of the tsetse following a bloodmeal, a temperature drop and an increase in acidity function cooperatively to induce differentiation from the BF to the PF. Together with remodeling of the parasite surface to replace VSG with procyclin, there are a number of cytoskeletal changes that occur, and the kinetoplast is repositioned. There is also a drastic change in metabolism as the trypanosomes leave the glucose-rich environment of the blood and transition to the fly midgut, where they are more reliant on mitochondrion Krebs cycle enzymes and respiratory chain and oxidative phosphorylation enzymes (reviewed in [5]).

Trypanosomes are unusual in that they are eukaryotes that transcribe their genome from polycistronic transcription units (PTUs) that often contain functionally unrelated genes. Thus, much regulation of gene expression occurs at the post-transcriptional level. With respect to differentiation, it is clear that the 3’UTRs of procyclin and *VSG* genes contribute to their developmental regulation [6–8], and that RNA binding zinc-finger proteins also play a role in specifying a differentiation program [9]. Many recent studies have shown that there are large changes in gene expression at the mRNA level during differentiation [10–12]. While developmental changes in gene expression have been examined at the level of mRNA stability and association with the translation machinery, the mechanisms by which these changes are mediated at the DNA level are not well understood. The fact that trypanosomes have recognizable histones, histone modifications, and orthologues of many of the chromatin-interacting proteins found in other, more familiar model systems made us wonder whether differentiation from the BF to the PF may be epigenetically regulated. As epigenetic modifications are reversible, this would confer the plasticity necessary to maximize fitness according to environmental input [13], allowing cells to be poised to move forward in development, but not until the receipt of the appropriate environmental signals. In mammals, bromodomain and extraterminal domain (BET) family members that recognize histone lysine acetylation through their bromodomains are necessary to maintain murine embryonic stem cell pluripotency [14,15], and differentiation can be rapidly induced by subjecting cells to treatment with highly selective bromodomain inhibitors [16]. Chromatin-interacting proteins in *T*. *brucei* have thus far been studied primarily in the context of monoallelic *VSG* expression and switching [17–20], but less is known about their potential role in lifecycle differentiation.

Here, we report that acetyl-lysine (AcK) recognizing bromodomain proteins maintain the BF cell fate in trypanosomes. Using both small molecule inhibitors and genetic mutants of individual bromodomain proteins, we demonstrate that bromodomain inhibition results in gene expression changes that mimic those seen naturally in cells differentiating from the BF to the insect stage following a bloodmeal and transition to the fly midgut. Transcriptional changes are recapitulated at the protein level by the appearance of insect-stage procyclin on the cell surface. BF-specific processes, including monoallelic expression of *VSG* genes and rapid surface-bound antibody internalization are also compromised, establishing *T*. *brucei* bromodomain proteins as attractive therapeutic targets for trypanosomiasis. Indeed, bromodomain inhibition decreases virulence in an in vivo mouse model. Our crystal structure of a trypanosome bromodomain in complex with an inhibitor revealed marked differences in the AcK recognition site to its human counterparts, consistent with a novel binding mode of the inhibitor, which can now be exploited to develop trypanosome-specific, high-affinity drugs against trypanosomiasis.

## Results

### Bromodomain Inhibition Increases Insect Stage-Specific Transcripts and Decreases Bloodstream-Specific Transcripts

**Fig 1:**
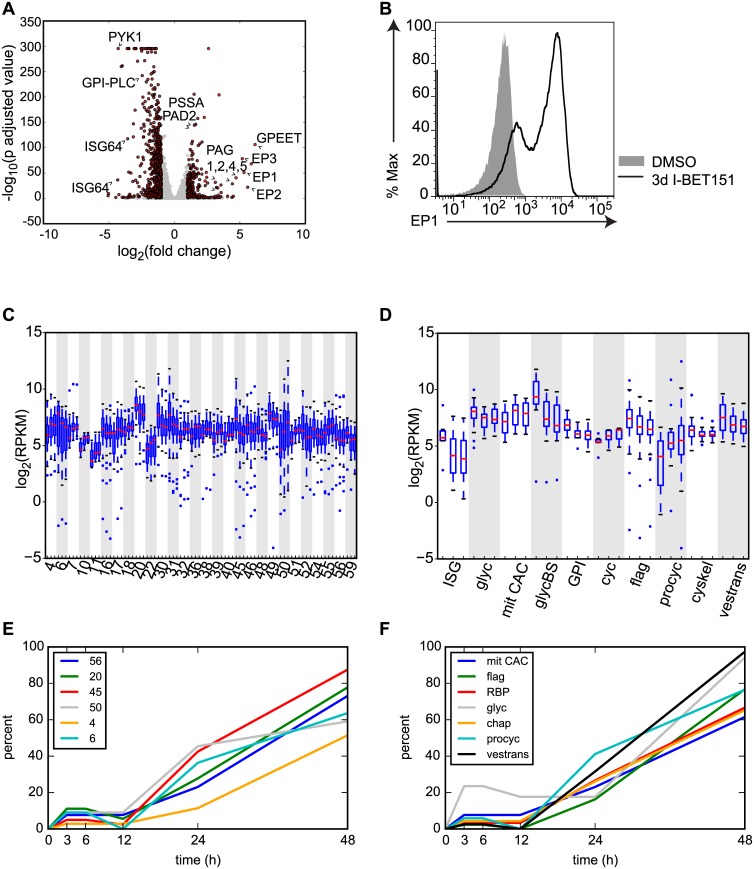
Trypanosome bromodomain proteins maintain a BF-specific transcriptome. (A) Volcano plot showing the most significant up- and down-regulated genes in the trypanosome genome from an RNA-seq experiment comparing cells treated for 3 d with I-BET151 to control DMSO-treated cells. DESeq was used to normalize counts, compute log_2_(fold change) of treated cells over untreated cells, and calculate *p*-adjusted values. Dots in red indicate genes with fold changes of > 2 and a *p*-adjusted value < 0.05. (B) Flow cytometry measuring surface EP1 expression in I-BET151-treated cells compared to control cells. (C) Boxplots showing log_2_(RPKM) values for all genes within hierarchically clustered groups previously shown to be differentially expressed in the transition from BF to PF [12]. Each cluster is demarcated by alternating white and gray shading and contains 3 boxplots representing DMSO-treated, 2d I-BET151-treated, and 3d I-BET151-treated cells. All clusters with a Gene Set Enrichment Analysis (GSEA) false discovery rate (FDR) < 0.1 and >70% of genes up- or down-regulated within each cluster at 48 h postinduction are shown. The median for each set of genes is shown as a red line. Whiskers demarcate the inner quartile range. Outliers are shown as blue dots. (D) Boxplots as in C except that the genes previously shown to be differentially expressed upon differentiation from the BF to the PF are now grouped in the indicated pathways. ISG, invariant surface glycoproteins, Glyc, glycosomal enzymes, mit CAC, mitochondrial citric acid cycle, glycBS, proteins required for high rate of glucose and glycerol metabolism in BFs, GPI, Glycosyl phosphatidylinosotol metabolism, cyc, cell cycle, flag, flagellar genes, procyc, procyclic surface antigens, cyskel, cytoskeletal genes, vestrans, proteins required for intracellular protein and vesicular transport. (E and F) DESeq was used to call genes with significantly altered transcription between I-BET151 treated cells and DMSO-treated control cells for every time point using a *p*-adjusted cutoff of < 0.1. For each functional group, the percentage of genes within that group that were significantly altered was calculated for each time point and plotted. Only groups with a GSEA FDR of < 0.1 are shown. (E) Early-induced hierarchically clustered gene sets. (F) Early-induced functional groups. chap, chaperone, RBP, RNA binding proteins. Numerical data for Fig 1 is found in S1 Data.

Bromodomain proteins have recently been implicated in the maintenance of stem cell pluripotency [14,15]. The *T*. *brucei* genome contains five genes with predicted bromodomains [21], and we hypothesized that these proteins may play similar roles in maintaining the parasites in their BF and preventing differentiation to the PF. To address this question, we took advantage of a highly selective small-molecule bromodomain inhibitor called I-BET151, a well-characterized successor of I-BET762, which inhibits BET bromodomain proteins in mammalian cells [22,23]. We performed RNA-seq experiments on I-BET151-treated cells and compared them to cells treated with a vehicle control (DMSO; dimethyl sulfoxide). For the initial dataset, cells were subjected to either two days or three days of treatment with I-BET151 and compared to DMSO-treated cells. The RNA-seq libraries were prepared in triplicate from three independent cultures for each treatment using polyA+ selection. We used DESeq analysis [24] to identify the top up-regulated and down-regulated genes following I-BET151 treatment. A list of these genes along with their associated multiple testing corrected *p*-adjusted values are listed in Additional_File_1 and Additional_File_2 in S1 Data. We used the log-fold changes and *p*-adjusted values from the DESeq analysis on I-BET151 cells treated for 3 d to generate the volcano plot shown in Fig 1A. Strikingly, I-BET151 treatment resulted in a strong up-regulation of a number of genes expressed only in the insect-residing PF form, including procyclins (*EP1-3* and *GPEET*), procyclin-associated genes (*PAG1*, *2*, *4*, *5*), procyclin stage surface antigen (*PSSA*), and protein associated with differentiation 2 (*PAD2*). We also observed a marked down-regulation of genes whose expression is normally higher in the mammalian BF, such as *ISG64*, *PYK1*, and *GPI-PLC* (Fig 1A). To confirm whether the up-regulation in transcription of *EP1* was reflected at the protein level, we performed flow cytometry experiments to ask whether EP1 was expressed on the surface of the cells. Indeed, a large proportion of I-BET151-treated trypanosomes expressed EP1 after 3 d of treatment, while this was not the case for vehicle-treated controls (Fig 1B). We conclude that bromodomain proteins are important for maintaining a BF-specific cell surface landscape.

Previous studies identified genome-wide changes in gene expression during differentiation from the BF to the PF and organized them both by hierarchical clustering of genes that varied with similar kinetics (clusters) and by functional group [12]. Each cluster contains a group of genes of both known and unknown function that were shown to change by roughly the same magnitude and with the same timing throughout differentiation. These genes are sometimes but not always functionally related. For example, about half the genes in cluster 30 are flagellar genes, but the remainder fall into different functional groups or have unknown function. Conversely, several flagellar genes are found in other clusters based on their pattern of expression. To analyze these same clusters of genes, we used a Gene Set Enrichment Analysis (GSEA) algorithm generated by the Broad Insitute at MIT [25] (http://www.broad.mit.edu/gsea). This algorithm generates an Enrichment Score that estimates the degree to which a given set of genes is over-represented within the total set of the most highly up-regulated or down-regulated genes. It also provides an Estimation of Significance level and corrects for multiple hypothesis testing to generate a false discovery rate (FDR). A list of the genes contained within each cluster is listed in Additional_File_3 in S1 Data and S12 Table. We analyzed our RNA-seq datasets comparing 2-d I-BET151-treated cells to control cells using GSEA enrichment with the clusters generated in [12]. S1 Table shows each cluster and its associated enrichment score and FDR for the 2-d I-BET151-treated dataset. To get a sense of how many of the genes within each cluster were moving coordinately in the same direction (up or down with respect to untreated cells), we conducted an additional analysis that determined the percent of genes up-regulated or down-regulated by 1.1-fold within each cluster in I-BET151-treated cells. S1 Table includes these percentages. We generated a list of clusters that had a GSEA generated FDR of <0.1 and for which > 70% of the genes were up-regulated or down-regulated following treatment with I-BET151. For the 31 clusters that met these criteria, we generated boxplots showing the expression level for all genes within that cluster in cells that had been treated for two days or three days with I-BET151, and compared them to control cells (Fig 1C). The red line on the boxplot indicates the median level of gene expression for all the genes within a cluster, while the whiskers denote genes falling within the inner quartile range. Outliers are shown as blue dots. In some cases, a progressive change was observed in the median level of gene expression for the cluster throughout the time course (e.g., 10, 20, 22). In other clusters, there was a shift in the distribution of the data in untreated versus 2-d-treated cells, but not between 2-d and 3-d-treated cells (e.g., 11, 46, 56). We conclude that gene expression changes that take place during differentiation for roughly half (31) of the clusters in the previous study were mirrored in I-BET151-treated cells.

Queiroz et al. also organized groups of genes that change throughout differentiation by their annotated function. These groups and their abbreviations are listed in Additional_File_4 in S1 Data and S12 Table. We performed the same analysis described above on these groups of genes and each functional group’s GSEA scores are listed in S2 Table. We generated boxplots for the ten functional groups that met our criteria for differential expression following treatment with I-BET151 (<0.1 GSEA FDR and >70% of genes up- or down-regulated)(Fig 1D, S2 Table). For example, during the transition from the BF to the PF, trypanosomes down-regulate genes required for high rates of glucose and glycerol metabolism (denoted as glycBS in Fig 1D) as they leave the glucose-rich environment of the blood and transition to the midgut of the fly. Other genes in the glycosomal pathway that are not involved in the pentose phosphate pathway are also down-regulated (denoted as glyc in Fig 1D). Cells undergoing differentiation also up-regulate many of the genes involved in the citric acid cycle (denoted as mit CAC in Fig 1D). We find these changes reflected in I-BET151-treated cells (Fig 1D, S2 Table). Notably, genes found in the procyclin-specific transcription unit (denoted as procyc in Fig 1D) were also up-regulated upon treatment with I-BET151 (Fig 1D, S2 Table). Other previously identified functional groups that met our criteria for differential expression in I-BET151-treated cells include the invariant surface glycoproteins (ISGs), genes involved in cell cycle (cyc), genes associated with glycosyl phosphatidylinositol metabolism (GPI), cytoskeletal genes (cyskel), flagellar genes, and genes associated with intracellular protein and vesicular transport pathways (vestrans) (Fig 1D). We conclude that with respect to transcription, bromodomain inhibited cells share some similarities to differentiating cells.

### A Temporal Analysis of I-BET151-Treated Cells

In order to compare the changes in gene expression that take place in I-BET151-treated cells with those that take place during differentiation, we performed a more extensive RNA-seq time course of I-BET151 treatment that included earlier time points than had been analyzed previously, at 3, 6, 12, and 24 h. As above, RNA-seq libraries were generated in triplicate using poly-A+ selection. First, we performed GSEA analysis using the Pearson metric for ranking genes in a time series (http://www.broadinstitute.org/gsea/doc/GSEAUserGuideFrame.html?_Metrics_for_Ranking). We thus obtained a list of 17 functional groups and 35 clusters that were over-represented in the set of genes that are differentially expressed through the time course using a GSEA FDR cutoff of <0.1 (S3 and S4 Tables).

We used DESeq’s negative binomial test to generate a list of differentially expressed genes at every time point using a multiple testing corrected *p*-adjusted cutoff of < 0.1. Differentially expressed genes and their associated expression and *p*-values for each time point are listed in Additional Files 5–9 in S1 Data. For those genes previously found to have altered gene expression during differentiation [12], we binned each DESeq generated differentially expressed gene into its associated cluster or functional group. We then plotted the percentage of genes within each cluster or group that were differentially expressed over the time course (Fig 1E and 1F, S1 and S2 Figs, S5 and S6 Tables, Additional Files 5–9 in S1 Data). Note that only the 17 functional groups and 35 clusters with a GSEA generated FDR of <0.1 (S3 and S4 Tables) are plotted in Fig 1E and 1F, S1 and S2 Figs. Note also that the percentages of differentially expressed genes used in the plots are in S5 and S6 Tables. For example, at 3 h after the initiation of I-BET151 treatment, 23% of genes in the glycosomal pathway (glyc, GSEA FDR = .018) are called by DESeq as being differentially expressed with a *p*-adjusted value of <0.1, whereas at 48 h, 94% of the genes in this group are differentially expressed in our dataset (gray line in Fig 1F, see also S5 Table). Other functional groups that showed early differential expression of their gene members include mitochondrial citric acid cycle enzyme genes (mit CAC), flagellar genes (flag), RNA binding protein genes (RBP), chaperone or protein-folding-related genes (chap), procyclic transcription unit genes (procyc) and genes associated with intracellular and vesicular transport pathways (vestrans) (Fig 1F).

The percentage of differentially expressed genes for each functional group rises sharply between 12 and 24 h for 15 of the groups analyzed (e.g., ISG and GPI, S1A Fig) (Fig 1F and S1A–S1C Fig) and generally continues to rise between 24 and 48 h (Fig 1F, S1B and S1C Fig). In addition to the ISG and GPI groups, genes involved in RNA degradation (RNAdeg), glucose and glycerol metabolism (glycBS), cytoskeleton(cyskel), ubiquitination (ubiq), DNA binding, replication, and nucleases (DNA) and protein kinase or kinase binding activity (signal K) were differentially expressed starting at 12 h after initiation of I-BET151 treatment (S1B and S1C Fig). Finally, two groups of genes were differentially expressed late in the time course, between 24 and 48 h. These included ribosomal proteins (ribprot) and genes associated with the nucleolus (nucleol) (S1D Fig).

We performed the same analysis described above on the 35 clusters with GSEA FDRs of <0.1. We found that six clusters had differentially expressed genes early in the time course (Fig 1E). 27 clusters contained genes that began to be differentially expressed at 12 h postinduction, and the percentage of differentially expressed genes within the majority of clusters continued to rise until 48 h (S2A–S2D Fig). Genes within Clusters 57 and 22 were not differentially expressed until 24 h postinduction (S2E Fig). Overall, this analysis indicates that a small subset of genes (between 21 and 29) are differentially expressed in the first 12 h of I-BET151 treatment, with a much larger number of genes (~700) showing differential expression between 12 and 24 h (see Additional Files 5–9 in S1 Data).

**Fig 2:**
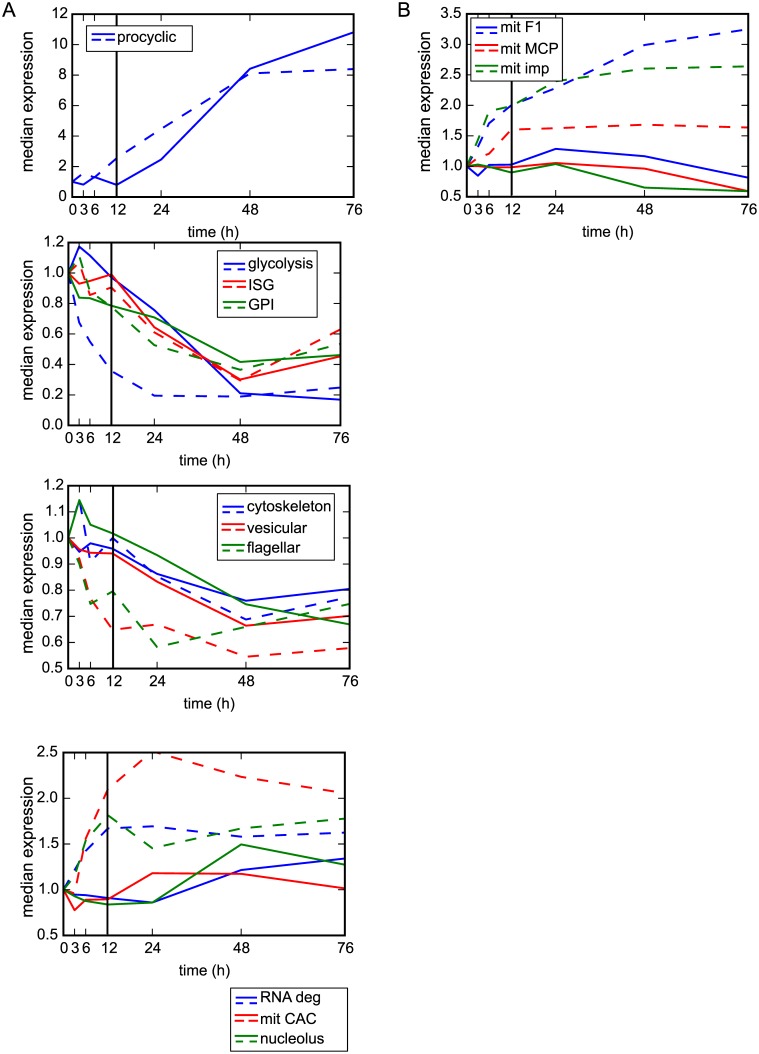
Transcriptional changes induced by I-BET151-treated cells mimic those induced by differentiating cells. (A) Plot showing median RPKM for all genes within the indicated functional groups at each time point after induction with I-BET151 (solid lines). For comparison, median expression values for all genes within the indicated functional groups were derived from data generated in [12] and plotted as dashed lines. Colors between solid and dashed lines are matched for each functional group. All functional groups with a GSEA FDR of <0.1 and where >70% of genes were up-regulated or down-regulated at 48 h of I-BET151 treatment are plotted. (B) Examples of 3 functional groups that do not match our criteria of a GSEA FDR of <0.1 and where >70% of genes were up-regulated or down-regulated at 48 h. mit F1, F1 ATPase, mit MCP, mitochondrial carrier proteins, mit imp, import of mitochondrial proteins. Numerical data for Fig 2 is in S2 Data.

We wanted to compare the timing for gene expression changes that took place during our I-BET151 RNA-seq time course with those that took place in the Queiroz study. We compared only those clusters and functional groups with a GSEA FDR of <0.1 and >70% of genes up- or down-regulated at the 48 h time point. To get a global picture of the expression changes within each cluster or functional group, we plotted the median reads per kilobase per million mapped reads (RPKM) for each cluster or group over time (Fig 2 and S3 Fig, solid lines). We artificially set the median for each group at time 0 to be 1 and normalized the other medians accordingly, in order to more easily compare changes between groups. We then calculated the median expression level for each cluster or group using data generated in the Queiroz study and plotted these medians alongside our data (Fig 2 and S3 Fig dashed lines). Each solid and dashed line was given the same color for each functional group or cluster to ease comparison between the studies. However, we would like to emphasize that caution must be taken when comparing these two datasets, because they were not only generated using different cells and different induction techniques, but also the previous study used microarray technology rather than sequencing technology. Thus the level of expression is only grossly comparable between the two datasets. Nevertheless, we did observe that in general, for the 10 functional groups that met our criteria (GSEA FDR of <0.1 and >70% of genes up- or down-regulated at the 48 h time point), the median gene expression between 12 and 76 h for each of the groups (to the right of the black line on each plot) roughly correlated with the previous study (Fig 2 and S3 Fig). While the median gene expression at 48 and 76 h was quite similar for some functional groups (e.g., procyclic, glycolysis, GPI, ISG), other functional groups in our dataset changed to a lesser extent than those in the previous study (e.g., mit CAC and RNA degradation) (Fig 2A). Also of note is that the expression changes were more poorly correlated between 3 and 12 h (to the left of the black reference line in Fig 2). For contrast, three functional groups that did not meet our criteria for similarity to the previous study (either > 0.1 GSEA FDR or <70% of group up- or down-regulated) are plotted in Fig 2B.

31 clusters matched our criteria for having a GSEA FDR of <0.1 and greater than 70% of genes up- or down-regulated (S3 Fig). In some cases, the median level of gene expression at 48 and 76 h matched quite well between the datasets (e.g., clusters 40, 6, 29, 54, 52, 51,10, 16, 39, and 31), while in other cases the changes in our dataset were more muted (e.g., clusters 32, 59, 17, 18). As with the functional groups, changes in gene expression between 3 and 12 h were more poorly correlated between datasets. Examples of clusters that did not pass our criteria for similarity to the previous study (either > 0.1 GSEA FDR or <70% of group up- or down-regulated) are plotted in S3B Fig.

For some clusters and functional groups analyzed (e.g., RNA deg, mit CAC, nucleolus, cluster 4,6,7), changes in gene expression as a result of I-BET151 treatment were delayed when compared to the gene expression changes that take place upon initiation of differentiation (Fig 2 and S3 Fig). We conclude that while I-BET151-treated cells show gross similarities to differentiating cells with respect to changes in the transcriptome, these changes are not identical for every group of genes, the changes are sometimes delayed, and in some cases the response is more muted.

### Inhibition of Bromodomain Proteins Disrupts BF-Specific Antibody Internalization

**Fig 3:**
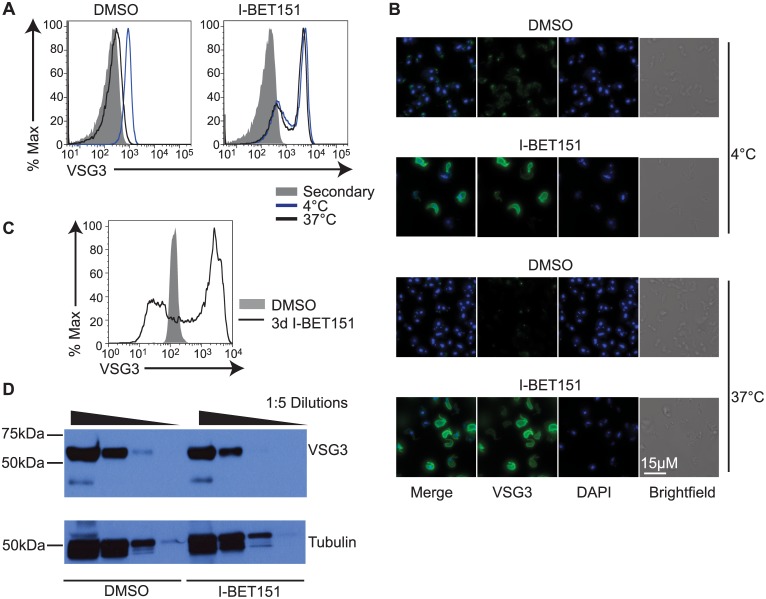
Bromodomain proteins are necessary for BF-specific antibody internalization. (A) Flow cytometry measuring the amount of primary anti-VSG antibody remaining on the surface following a 5-min incubation at the indicated temperatures, washout, fixation, and incubation with secondary antibody in I-BET151-treated (right) versus control cells (left). "Secondary" (gray trace) denotes control cells stained with secondary antibody only. (B) Immunofluorescence showing the amount of primary anti-VSG antibody remaining on the surface following a 5-min incubation at the indicated temperatures, washout, fixation, and incubation with secondary antibody in I-BET151-treated versus control cells. A bright spot is detected at the flagellar pocket in control cells incubated at 4°C, whereas I-BET151-treated cells have antibody distributed over the entire surface. At 37°C, control cells have internalized almost all the primary antibody. (C) Flow cytometry showing the amount of anti-VSG3 antibody bound to the surface in cells treated with I-BET151 for 3 d, compared to control cells. (D) Anti-VSG Western blot showing that protein levels of *VSG* at the active expression site (ES) are unaltered in I-BET151-treated cells. Tubulin is used as a loading control. Numerical data for Fig 3 is in S3 Data.

Genes with functions in the intracellular protein transport and vesicular transport pathways are necessary for VSG recycling and are down-regulated during differentiation [26]. Consistent with this observation, we found that genes within the vesicular and intracellular transport group were down-regulated in I-BET151-treated cells (Figs 1D and 2A and Additional Files 1,2, and 5–9 in S1 Data). Concurrently, expression of genes required for motility and for VSG-bound antibody internalization [4] are down-regulated during differentiation and were also reduced in I-BET151-treated cells (denoted “flag” in Fig 1D and flagellar in Fig 2A). We therefore tested whether I-BET151-treated cells were able to internalize surface-bound antibody. We first coated the cells with a primary antibody against the expressed surface VSG. After washing unbound antibody away, we subjected the cells to a 5-min incubation at 4°C or 37°C. At 4°C, wildtype cells are unable to move and the antibody remains on the surface, whereas at 37°C they are able to move freely and internalize surface-bound antibody [4]. Following incubation, we immediately fixed the cells with formaldehyde. We then added a secondary antibody and measured how much primary antibody remained on the surface by flow cytometry and immunofluorescence. We observed a complete block in antibody internalization in I-BET151-treated cells (Fig 3A and 3B), consistent with decreased motility observed in these cells (compare S1 and S2 Movies). We also observed an increase in the amount of anti-VSG antibody bound to the surface in I-BET151-treated cells (Fig 3C) without a concomitant increase in total VSG protein (Fig 3D). We attribute this accumulation in surface-bound antibody to the block in antibody internalization and possible defects in VSG recycling associated with down-regulation of many of the genes necessary for these processes (Figs 1D and 2A). These data strongly suggest that bromodomain proteins are necessary for maintaining the BF cell fate, and that their inhibition has functional consequences on BF-specific immune evasion processes, such as rapid internalization of surface-bound antibodies.

### Bromodomain Proteins Are Necessary for Monoallelic Expression of *VSG* Genes

Recent work has demonstrated a mechanistic link between *VSG* expression and differentiation from BF to PF cells [27]. *VSG*s are located in metacyclic-specific expression sites (metacyclic expression sites [ESs]), minichromosomes, and elsewhere in the megabase chromosomes (S4A Fig). In the BF, they are transcribed from one of ~15 telomeric ESs, but only one ES is transcriptionally active at any one time. Each ES contains a promoter and a number of *Expression Site Associated Genes* (*ESAG*s) upstream of the *VSG*. Batram et al. discovered that ectopic expression of a *VSG* gene causes silencing to spread from the active ES telomere upstream toward the promoter, and that this silencing “primes” cells to transition from the BF to the PF. We used RNA-seq to ask whether a similar phenomenon occurs in I-BET151-treated cells by mapping reads that did not align to the megabase chromosomes to ES *ESAG*s and to *VSG*s [28]. We observed an increase in transcription of silent *VSG*s genome-wide (S4B Fig, Additional File 10 in S10 Data). Q values for the VSGs were calculated by subjecting *p*-values generated from the replicate sets to Benjamimi and Hochberg correction using SeqMonk software from Babraham Bioinformatics (see Materials and Methods) and VSGs with a Q value of < 0.1 are shown in red in S4B Fig. Examples of cells expressing more than one VSG could be observed by staining I-BET151-treated cells with multiple VSG antibodies and using an Imagestream flow cytometer to visualize double-expressing cells (S4C Fig, top). However, it should be noted that the proportion of cells with two different VSGs on the surface is <1% of the total population (S4C Fig, bottom), and thus the loss of monoallelic expression appears to take place mainly at the transcriptional level.

We hypothesized that the increase in VSG mRNA could result in active ES attenuation, as was observed by Batram et al. A decreasing transcription gradient at the active ES has also been demonstrated at the beginning of differentiation from the BF to the PF [29]. To unambiguously map the *ESAG* reads to each ES, we aligned the reads uniquely allowing for 0 mismatches. This analysis revealed an increase in transcription of *ESAG*s near the active ES promoter, while those near the active ES telomere were silenced, with silencing spreading upstream toward the promoter between 2 and 3 d of treatment (S4D Fig, Additional File 11 in S10 Data). These results support the notion that I-BET151-treated cells are primed for differentiation. Another link between ES expression and differentiation was observed by Amiguet-Vercher et al., who found an increase in transcription in regions near the ES promoter in silent ESs during the early stages of differentiation [29]. We also observed an increase in transcription of *ESAG*s at silent ESs upon treatment with I-BET151 (S4E Fig). We plotted the median log_2_(RPKM) for all *ESAG*s in silent ESs during the I-BET151 time course and found an initial increase in expression of these *ESAG*s at 3 h postinduction, with a much more dramatic increase in expression occurring between 24 and 48 h (S5 Fig). To ascertain the timing for increased expression of silent VSGs, we plotted the median log_2_(RPKM) for all inactive VSGs during our time course and observed that VSG expression rises later in the time course, primarily between 24 and 48 h (S5 Fig). We conclude that bromodomain proteins are necessary for maintaining monoallelic expression of VSG genes, and confirm that transcription of *ESAG*s and *VSG*s appears intimately linked to differentiation processes.

### Bromodomain Proteins Mediate "Memory" of the Active ES

**Fig 4:**
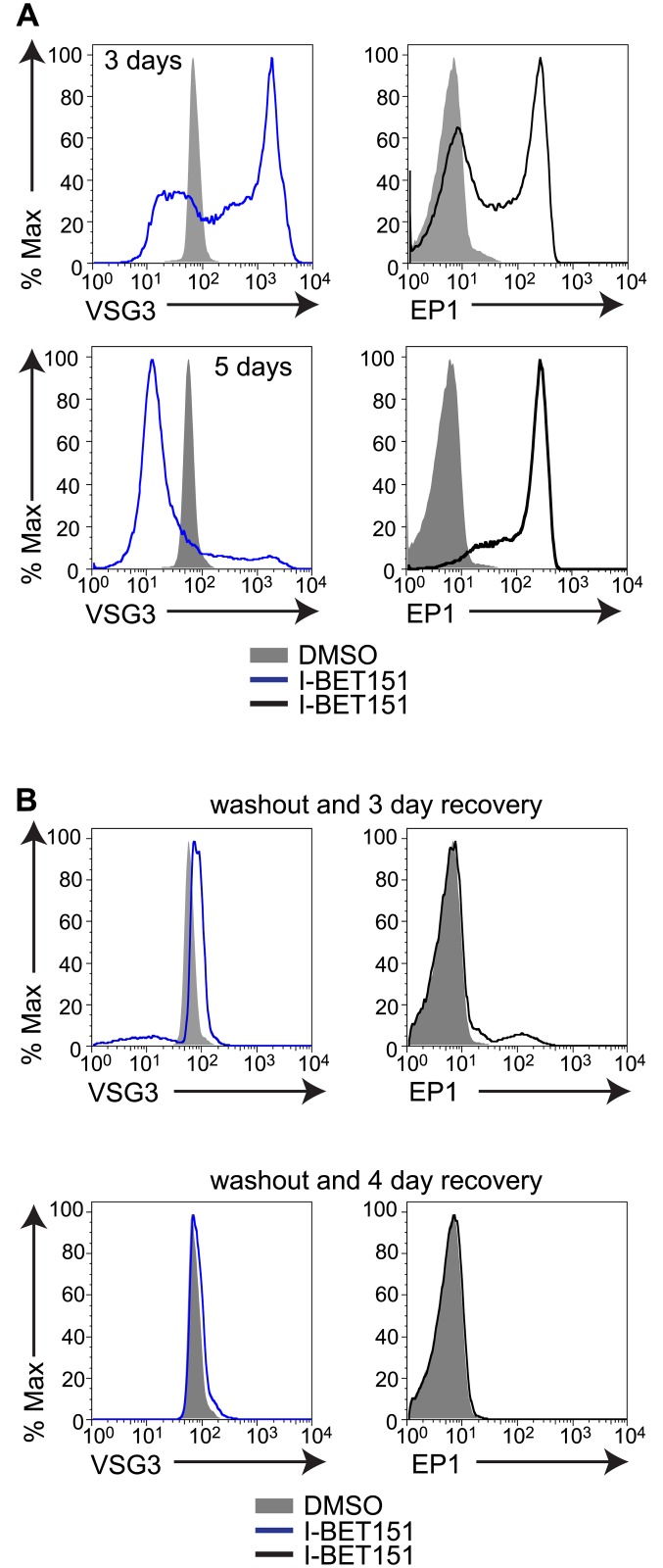
Bromodomain inhibition is reversible and “memory” of the active ES is retained. (A) Flow cytometry measuring the amount of anti-VSG antibody bound to the cell surface as well as surface levels of EP1 following treatment with I-BET151 for the indicated number of days. (B) Flow cytometry measuring surface levels of VSG and EP1 following a 5-d treatment with I-BET151, washout, and recovery for 3 d (top two panels) or 4 d (bottom two panels). Numerical data for Fig 4 is in S4 Data.

In mammalian cells, differentiation from a totipotent to a differentiated state is accompanied by a number of changes at the chromatin level, including deposition of repressive histone marks and increasing chromatin compaction. Cellular reprogramming to the undifferentiated state is characterized by a nucleus largely devoid of heterochromatin [30], and is inhibited by repressive histone modifications [31]. While mammalian cells generally remain in their differentiated state, trypanosomes are required to cycle continuously between alternate differentiated states, and thus the epigenetic changes that accompany these transitions must necessarily retain some degree of plasticity. In agreement with this notion, the effects of I-BET151 treatment are reversible. Longer treatments (5 d) with I-BET151 lead to very low levels of anti-VSG antibody bound to the cell surface (Fig 4A), but memory of the active ES appears to be retained, as the VSG associated with this same ES is expressed on the surface after 3 d of washing out the drug (Fig 4B). Similarly, levels of surface EP1 are high after 5 d of I-BET151 treatment, but expression is lost upon washout of the drug. These results imply that the transcription changes associated with differentiation are reversible, and supports the intriguing possibility that these changes may be epigenetically mediated, though this remains to be conclusively proven.

### Genetic Ablation of Bromodomain Proteins Phenocopies Treatment with I-BET151

**Fig 5:**
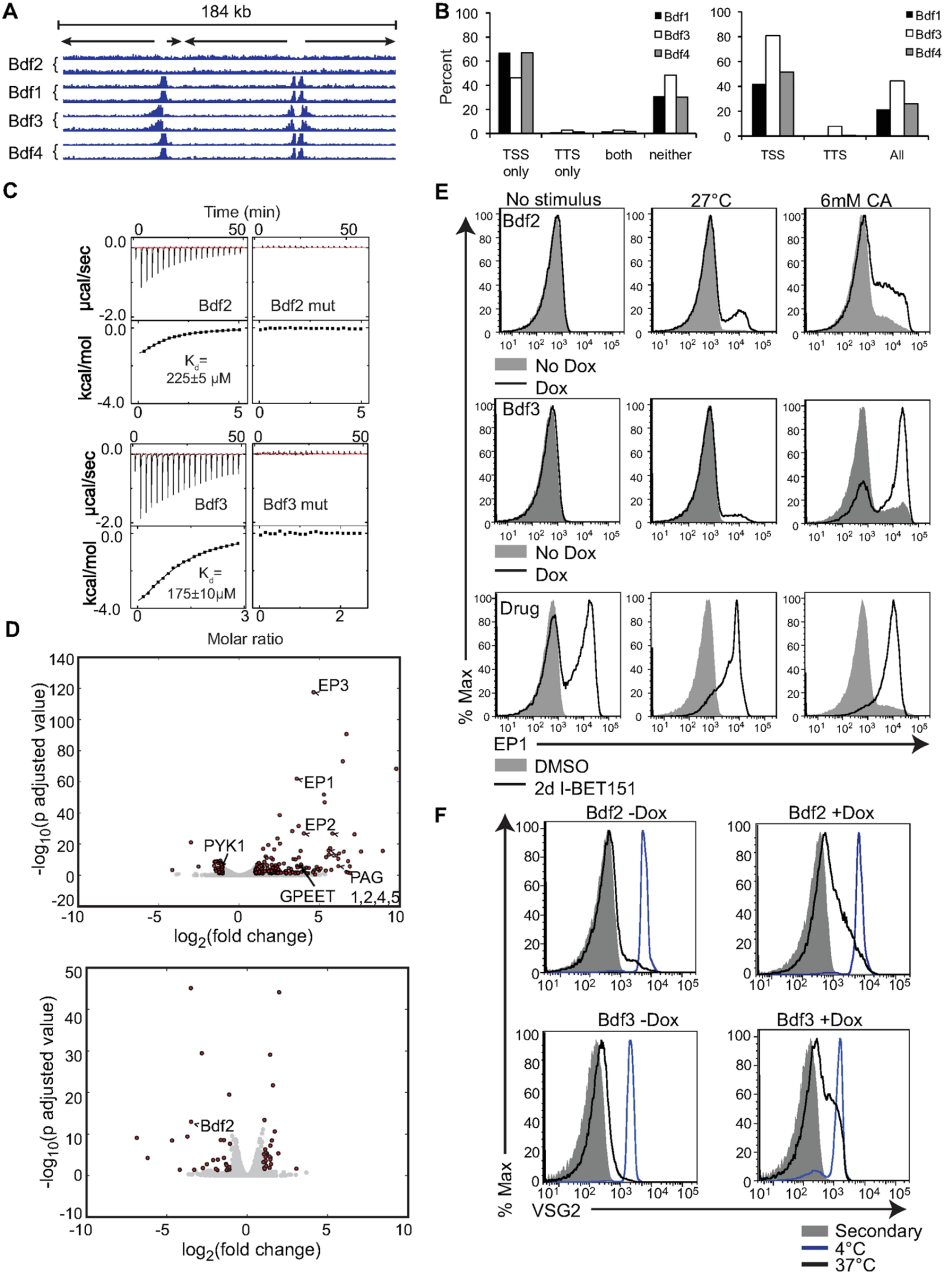
Genetic depletion of bromodomain proteins phenocopies treatment with I-BET151. (A) Left, Chromatin immunoprecipitation and sequencing (ChIP-seq) of four endogenously HA-tagged trypanosome bromodomain proteins showing localization to sites of transcription initiation for three of them. The region shown is a 184 kb region from chromosome 7. Arrows represent direction of transcription for PTUs. Areas where the arrows diverge are sites of transcription initiation. (B) Left, the percent of all MACS-derived peaks lying within 100 bp of a divergent strand switch region (Transcription Start Site, TSS), convergent strand switch region (Transcription Termination Site, TTS), both (a peak that is within a 100 bp of both a TSS and a TTS) or neither, a peak that is >100 bp away from an TTS or TSS. Right, the percent of all transcription start sites within 100 bp of a peak are shown for Bdf1,3,4. No Bdf2 MACS-derived peaks were within the FDR < 0.1 cutoff and are thus not included in this analysis. The total number of peaks called for Bdf1, 3 and 4 are 240, 307, and 175 respectively. Note that “head to tail” sites were not included in this analysis. (C) Isothermal titration calorimetry measuring binding of Bdf2 and Bdf3 to I-BET151. Conserved tyrosine and asparagine residues within the AcK binding pocket are mutated to alanine in the mutant constructs. (D) Volcano plot showing the most significant up-regulated and down-regulated genes in the trypanosome genome from an RNA-seq experiment comparing Dox-treated Bdf3^kd^ (top) or Bdf2^KO^ (bottom) to untreated control cells. DESeq was used to normalize counts, compute log_2_(fold change) of treated cells over untreated cells, and calculate *p*-adjusted values. Dots in red indicate genes with fold changes of > 2 and a *p*-adjusted value < 0.05. (E) Flow cytometry measuring surface EP1 expression in two-d Dox-treated Bdf2^KO^ (top) or Bdf3^kd^ cells (middle), or I-BET151-treated cells (bottom) following washout of IBET-151 or Dox, and subsequent exposure to a 24 h treatment with one of two different differentiation triggers: incubation at 27°C (middle) or treatment with cis-aconitate (CA) at 37°C (right) compared to a control without any trigger (left, No stimulus). (F) Flow cytometry measuring the amount of primary anti-VSG antibody remaining on the surface following a 5-min incubation at the indicated temperatures, washout, fixation, and staining with secondary antibody in Dox-treated Bdf2^KO^ (top panels) and Bdf3^kd^ cells (bottom panels) compared to controls. Numerical data for Fig 5 is in S5 Data, except wiggle plots shown in Fig 5A, which are in S6 Data.

We then set out to confirm that the observations associated with I-BET151-treated cells are mediated by the inhibition of trypanosome bromodomain proteins. First, we endogenously tagged Bdf1-4 with C-terminal HA (Bdf5 function has already been studied [32,33]). We performed chromatin immunoprecipitation-sequencing (ChIP-seq) experiments and used the Model-based Analysis for ChIP-Seq (MACS) algorithm to determine peaks of localization for each tagged Bdf [34]. Each experiment was performed in duplicate, and only those peaks that were called by the MACS algorithm in both replicates were analyzed. Each peak was required to have a MACS algorithm-based FDR of <0.1 in at least one replicate to be included in the analysis presented in Fig 5B. Additionally, we called peaks in control samples that were subjected to anti-HA pull downs but that did not contain tagged proteins. Any peak that was called by MACS in these control lines was removed from the set of called peaks in each of the tagged lines. Genomic location, FDR values, and fold enrichment for each called peak are in Additional Files 12–14 in S5 Data. We confirmed binding of Bdf3 to sites of transcription initiation [35] and found that Bdf1 and 4 also localized to these sites, while Bdf2 did not (Fig 5A). Specifically, 67%, 46% and 67% of Bdf1, 3, and 4 peaks, respectively, are within 100 bp of a divergent strand switch region (with two PTUs going in opposite directions, presumed to be transcription start sites) (Fig 5B, left). 52%, 85% and 70% of all divergent strand switch regions (Transcription Start Sites, TSSs) are within 100 bp of a Bdf1, 3, or 4 peak, respectively (Fig 5B, right). In contrast, although the MACS algorithm did call 71 Bdf2 peaks in the genome, none of them met the <0.1 FDR cutoff, and were thus not included in this analysis. Based on genomic localization, we divided bromodomain proteins into two classes: those that bind to transcription start sites (Bdf1, 3, 4) and those that do not (Bdf2). We decided to further study Bdf3 and Bdf2 as representatives of each class.

Using the recombinantly expressed and purified bromodomains from *T*. *brucei* Bdf2 and Bdf3 (Tb927.10.7420, Tb927.11.10070, respectively), we performed isothermal titration calorimetry experiments and confirmed direct and specific binding of I-BET151 to their AcK recognition sites, as mutation of two conserved residues in the AcK sites abolished interaction with the inhibitor (Fig 5C). By contrast, we could not detect binding of another well characterized bromodomain inhibitor, JQ1 [36], to recombinant Bdf2 and Bdf3 bromodomains (S6 Fig), which is consistent with the fact that JQ1 had no biological effects on trypanosomes in our hands (S12 Data). Having confirmed Bdf2 and Bdf3 as I-BET151 targets, we next asked whether genetic ablation of these proteins would phenocopy the effects of treating trypanosomes with I-BET151.

To enable functional characterization of Bdf2, we created a conditional knockout strain with a floxed, endogenously HA-tagged allele of *Bdf2* (Bdf2^KO^ cells) and replaced the remaining allele with a floxed drug resistance marker. Doxycyclin (Dox) treatment induces Cre expression to delete floxed alleles of *Bdf2* in the Bdf2^KO^ strain. To study the function of Bdf3, we created a separate, inducible RNAi strain harboring an endogenously HA-tagged *Bdf3* (Bdf3^kd^ cells). Here, Dox treatment induces expression of double-stranded RNA complementary to *Bdf3* (S7A Fig). Dox treatment of Bdf2^KO^ and Bdf3^kd^ cells resulted in efficient protein knockdown of Bdf2 and Bdf3, respectively (S7B and S7C Fig). We observed normal growth in the Bdf2^KO^ cells until 48 h after induction, where a growth defect emerged, coinciding with perturbations in cell cycle (S8A and S8C Fig). Bdf3^kd^ cells were growth arrested between 12 and 24 h, and we observed cell cycle defects at 24 h (S8B and S8C Fig). Cell cycle defects largely resolved between 1 and 2 d of treatment (S8C Fig, right panel, coinciding with a restoration in Bdf3 protein level at 48 h (S8D Fig, bottom), implying that the RNAi against Bdf3 appears to shut off after 24 h by an unknown mechanism.

RNA-seq analysis was performed on Bdf2^KO^ and Bdf3^kd^ cells using similar methods as those described for I-BET151 treatment. Because the kinetics for deletion of Bdf2 were slightly slower than for knockdown of Bdf3 (S8D Fig), we allowed a longer time period for induction of Bdf2 deletion and associated downstream transcriptional effects prior to harvesting RNA (48 h for Bdf2 versus 24 h for Bdf3 knockdown). polyA+ RNA was isolated from 3 independent biological samples to generate RNA-seq libraries for cells treated with and without Dox. DESeq analysis revealed that many of the insect-specific genes that were up-regulated in I-BET151-treated cells were also up-regulated in Bdf3^kd^, but not in Bdf2^KO^ cells, including *EP1-3*, *GPEET2* and procyclin-associated genes *PAG1*, *2*, *4*, *5* (Fig 5D, Additional File 15 and Additional File 16 in S5 Data). We performed GSEA analysis in Bdf3^kd^ and Bdf2^KO^ cells using the same clusters and functional groups described earlier, which had previously been shown to be differentially expressed during the transition from the BF to the PF [12]. All results of GSEA analysis on Bdf3^kd^ and Bdf2^KO^ cells are listed in S7–S10 Tables. Of the functional groups analyzed, 10 groups met our criteria of having a GSEA FDR of <0.1 and 70% of genes moving in one direction in Bdf3^kd^ cells (S7 Table). Boxplots of these functional groups are shown in S9A Fig, left. By contrast, only one functional group passed our criteria in Bdf2^KO^ cells (S9B Fig, left). Of the clusters analyzed, six clusters passed our criteria in Bdf3^kd^ cells (S9A Fig, right), while four clusters passed in Bdf2^KO^ cells (S9B Fig, right).

RNA-seq analysis revealed that transcripts for VSGs located at silent bloodstream expression sites (BESs) were significantly increased in both Bdf3^kd^ and Bdf2^KO^ cells. This was also true for metacyclic *VSG*s, while *VSG*s in minichromosomes were only slightly up-regulated in Bdf3^kd^ cells (S10A Fig). Consistent with its more widespread genomic localization, knockdown of Bdf3 increased transcription for more of the *VSG*s in the genome than was the case for Bdf2 (S10B Fig). These results imply that while both proteins maintain monoallelic expression of *VSG* genes, Bdf2 has a more specialized role in maintaining silencing at ESs. We tested whether Bdf2 could be localized to the ES using chromatin IP followed by Q-PCR. We performed this test in a cell line with a Blasticidin resistance gene at the active ES promoter and a GFP marker at one of the silent ES promoters. We found that Bdf2 localized to the promoter region of both the active and silent ES, and we also detected weaker binding at each of the *VSG*s associated with these ESs (S10C Fig, left). Binding to these regions was decreased in the presence of I-BET151 (S10C Fig, right). We also tested binding of Bdf3 to the ES using a cell line that did not distinguish the promoters of active and silent ESs. We found that Bdf3 also binds to the ES promoter (ESP1 in S10D Fig) and to a pseudogene located further down the ES, but that it did not appear to bind directly to the *VSG* genes (S10D Fig). Finally, transcription of *ESAGs* in inactive ESs is also higher in Bdf3^kd^ and Bdf2^KO^ cells (S11 Fig).

We further tested whether Bdf3 binding was altered in the presence of I-BET151 by collecting samples for ChIP at 6 h, 12 h, and 24 h following initiation of treatment. We then used Q-PCR to test whether Bdf3 binding was altered at the ES promoter (ESP1 in S12A Fig) or at several other sites identified as peaks of Bdf3 binding in our ChIP-seq experiment (S12B–S12D Fig). We found that I-BET151 treatment decreased binding to both the ES promoter and to the other sites we tested in a progressive manner, whereas it did not decrease binding to the *URA3* control region (S12A Fig).

Our RNA-seq experiments on Bdf2^KO^ and Bdf3^kd^ cells were conducted at 48 h and 24 h post-induction, respectively. We wanted to confirm that the changes in transcription at these later time points were reflected early upon depletion of each Bdf. To do this, we isolated RNA from Bdf2^KO^ cells at 6, 12, 24, 36, and 48 h postinduction with Dox. For Bdf3^kd^ cells, we isolated RNA at 6, 12, and 24 h postinduction. We confirmed that PAG2, EP1, and GPEET2 mRNA expression increased after 6 h of induction in Bdf3^kd^ cells. This was also the case for a *VSG* gene in a silent site (VSG3) (S13A Fig). We were also able to confirm that the transcription of silent ES VSGs and one metacyclic VSG was increased in Bdf2^KO^ cells as early as 6 h postinduction (S13B Fig).

Genetic depletion of Bdf3 did not result in the expression of EP1 on the surface of the cells (Fig 5E), but previous results from Batram et al. indicated that the presence of an ectopically expressed VSG “primed” cells for differentiation from the BF to the PF and increased their sensitivity to cis-aconitate, an acidic small-molecule differentiation trigger that mimics the increased acidity of the fly midgut [27,37,38]. We wondered whether the increase in transcription of normally silenced *VSG*s might have a similar effect, so we subjected Bdf3^kd^ and Bdf2^KO^ cells to treatment with cis-aconitate or reduced temperature. Indeed, genetic depletion of bromodomain proteins primes BF trypanosomes for differentiation, and requires only one (cis-aconitate), rather than two, environmental stimuli to induce EP1 expression (Fig 5E). The effect was stronger in Bdf3^kd^ cells, perhaps due to the significant up-regulation of EP1 mRNA. Unlike the Bdf2- and Bdf3-depleted trypanosomes, a large proportion of I-BET151-treated cells expressed EP1 at high levels in the absence of any environmental stimulus, and nearly all cells expressed EP1 following treatment with cis-aconitate or incubation at 27°C (Fig 5E). Control cells with intact bromodomain proteins showed very low induction of EP1 upon exposure to differentiation triggers (Fig 5E). These results confirm that bromodomain proteins are important for preventing remodeling of the parasite surface until all the appropriate environmental signals have been received.

We were able to partially recapitulate the block in antibody internalization observed in I-BET151-treated cells in both Bdf3^kd^ and Bdf2^KO^ cells (Fig 5F). The block in Bdf3^kd^ cells is consistent with the decrease in transcription of flagellar genes necessary for motility (S9 Fig). The partial block in antibody internalization observed in Bdf2^KO^ cells was unexpected, but might be explained by a decrease in expression of the paraflagellar rod gene *Pfr2* (Tb927.8.5010) and in α-tubulin (Tb927.1.2400) in the Bdf2^KO^ RNA-seq dataset. We hypothesize that these might be indirect consequences of Bdf2 inhibition related to *VSG* derepression [27]. Notably, the effects of I-BET151 treatment were more pronounced than those in cells with depletion of individual bromodomains, which most likely reflects simultaneous inhibition of at least two bromodomains (Bdf2 and Bdf3) by I-BET151. We cannot rule out the possibility that I-BET151 may also bind Bdf1, 4, and/or 5, and that these proteins may be able to partially compensate for the lack of Bdf3 in Bdf3^kd^ cells. Overall, we show that most of the effects seen in I-BET151-treated cells are phenocopied in Bdf3^kd^ cells, and that ES-specific effects are also observed in Bdf2^KO^ cells. Our results imply that bromodomain proteins are required for maintenance of BF stage identity.

### Bromodomain Proteins Are Promising Drug Targets for Treatment of Trypanosomiasis

**Fig 6:**
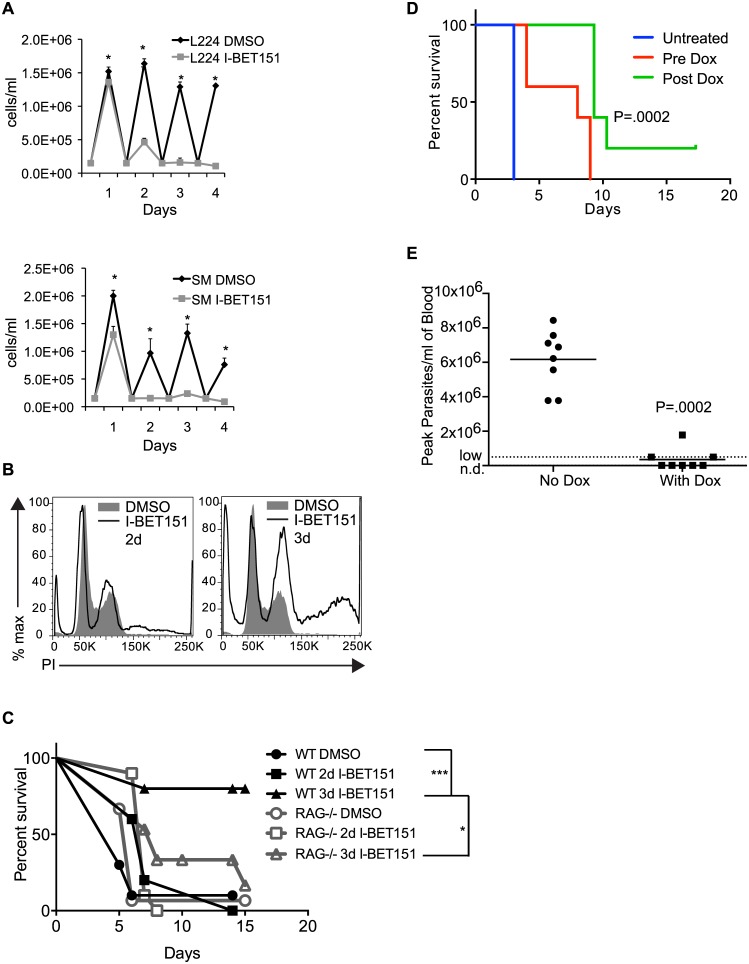
Bromodomain inhibition decreases virulence in a mouse model of infection. (A) Growth curves for two different strains used in the study (L224 and SM). I-BET151-treated cells were incubated at 37°C and counted daily on a hemacytometer and parasite numbers were compared with control cells. * indicates a *t*-test *p*-value of < 0.1. (B) Flow cytometry of propidium iodide (PI)-stained cells to measure DNA content following treatment with I-BET151 compared to controls. (C) Survival curves of C57/B6 or *RAG-/-* mice infected with trypanosomes pretreated in vitro with I-BET151 or DMSO (vehicle control) for the indicated number of days. * indicates significant *p*-value of < .05 derived from Log-rank Mantel-Cox test between the indicated groups. *** indicates significant *p*-value of < .001 derived from Log-rank Mantel-Cox test between the indicated groups. Note that one *RAG-/-* mouse was sacrificed at day ten due to infection unrelated to trypanosomiasis. (D) Survival curves of C57/B6 mice infected with Bdf3^kd^ trypanosomes and fed with Dox pellets for 2 d prior to infection (Pre Dox), or once parasitemia reached detectable levels (Post Dox) compared with mice fed with regular pellets. *p*-Value derived from Log-rank Mantel-Cox test performed on the three groups. (E) Peak levels of parasites/ml of blood measured by tail bleed in Dox-fed wildtype mice infected with Bdf2^KO^ trypanosomes compared to mice fed with regular pellets. *p*-Value derived from Mann-Whitney test. Numerical data for Fig 6 is in S6 Data.

Inhibition of bromodomain proteins compromises BF-specific immune evasion mechanisms and provides several advantages for the host immune system, including slower parasite growth and cell cycle defects (Fig 6A and 6B and S8 Fig), expression of an invariant epitope on the surface of the cell (EP1) (Fig 1B), and defects in antibody internalization (Figs 3A and 5F), making these proteins attractive drug targets. However, while repurposing known bromodomain inhibitors to combat trypanosomiasis is a promising strategy, in vivo treatment with I-BET151 itself is suboptimal because of its low affinity for trypanosome bromodomain proteins and its high affinity for mammalian BET proteins that affect the immune system [22]. We therefore asked whether the course of parasitemia is altered in mice infected with trypanosomes treated in vitro with I-BET151 prior to infection. Mice infected with parasites treated for two days with I-BET151 survived significantly longer than control mice. In addition, 80% of the mice infected with parasites treated for three days with I-BET151 did not develop detectable parasitemia (Fig 6C). We wanted to make sure that the survival advantage in WT mice was not due to lack of viability of I-BET151-treated cells, and we also wanted to confirm that I-BET151-treated cells were able to establish infections in vivo. We reasoned that if the drug-treated trypanosomes were not viable or capable of establishing an infection, then *RAG-/-* mice, which lack B and T cells, would have a similar advantage to WT mice in surviving infection with I-BET151-treated trypanosomes. We thus performed the same experiment in *RAG-/-* mice and observed that survival was significantly lower in *RAG-/-* mice that had been treated with I-BET151-treated trypanosomes when compared to WT mice (Fig 6C). We conclude that the survival advantage conferred by I-BET151-treated cells in WT mice cannot be solely due to lack of viability of drug-treated trypanosomes, since they are capable of killing immunocompromised mice.

We performed two additional control experiments to ensure that drug-treated trypanosomes were viable in vitro. We treated trypanosomes with I-BET151 or DMSO for 3 d and then washed out the drug. We plated the treated cells in 3 separate 96-well dishes at a concentration of 50 cells/well and then counted the number of wells where trypanosomes were growing after 4 d. We chose this concentration because we infected mice with 50 trypanosomes. 100% of wells in all 3 plates had trypanosomes growing after 4 d for both DMSO and I-BET151-treated samples (S14A Fig). This is not to say that there is no loss in viability; only 66% of wells with I-BET151-treated cells plated at a concentration of 5 cells/well grew into a colony after 4 d (as opposed to 100% of WT cells) (S14A Fig). Finally, we used flow cytometry to measure the percent of propidium iodide (PI)+ cells following 3 d of treatment with I-BET151 and again after washout and recovery. We found that while the percent of PI+ cells in the population was 3.5 times higher in I-BET151-treated cells compared to DMSO-treated cells, the percentage of PI+ cells in the I-BET151-treated population was only 1.1% overall (S14B Fig). The percent of PI+ cells after 3 d of washout and recovery was 0.3% for both populations (S14B Fig). We conclude that it is highly probable that there were live cells within the 50 cell starting population of I-BET151-treated cells used to infect the mice for the experiments shown in Fig 6. Consistent with the data in Fig 6A, we did observe a lag in growth in I-BET151-treated cells, both prerecovery and postrecovery, which might contribute to the increased survival of WT mice infected with I-BET151 treated trypanosomes (S14C Fig).

We also tested the effects of genetically depleting bromodomain proteins in vivo. We infected mice with Bdf3^kd^ cells and knocked down Bdf3 in vivo by feeding infected mice with Dox pellets either before or after parasitemia became detectable. We observed an increase in survival specifically in Dox-fed mice (Fig 6D). Mice infected with *Bdf2* conditional knockout parasites were able to clear the infections without Dox treatment, possibly due to leakiness of the *Cre* gene and lower levels of Bdf2, compromising growth. Nonetheless, mice fed with Dox pellets had extremely low or undetectable parasitemia when compared with regular pellet-fed mice (Fig 6E).

### The Crystal Structure of Bdf2 in Complex with I-BET151 Reveals a Novel Binding Mode of the Inhibitor

**Fig 7:**
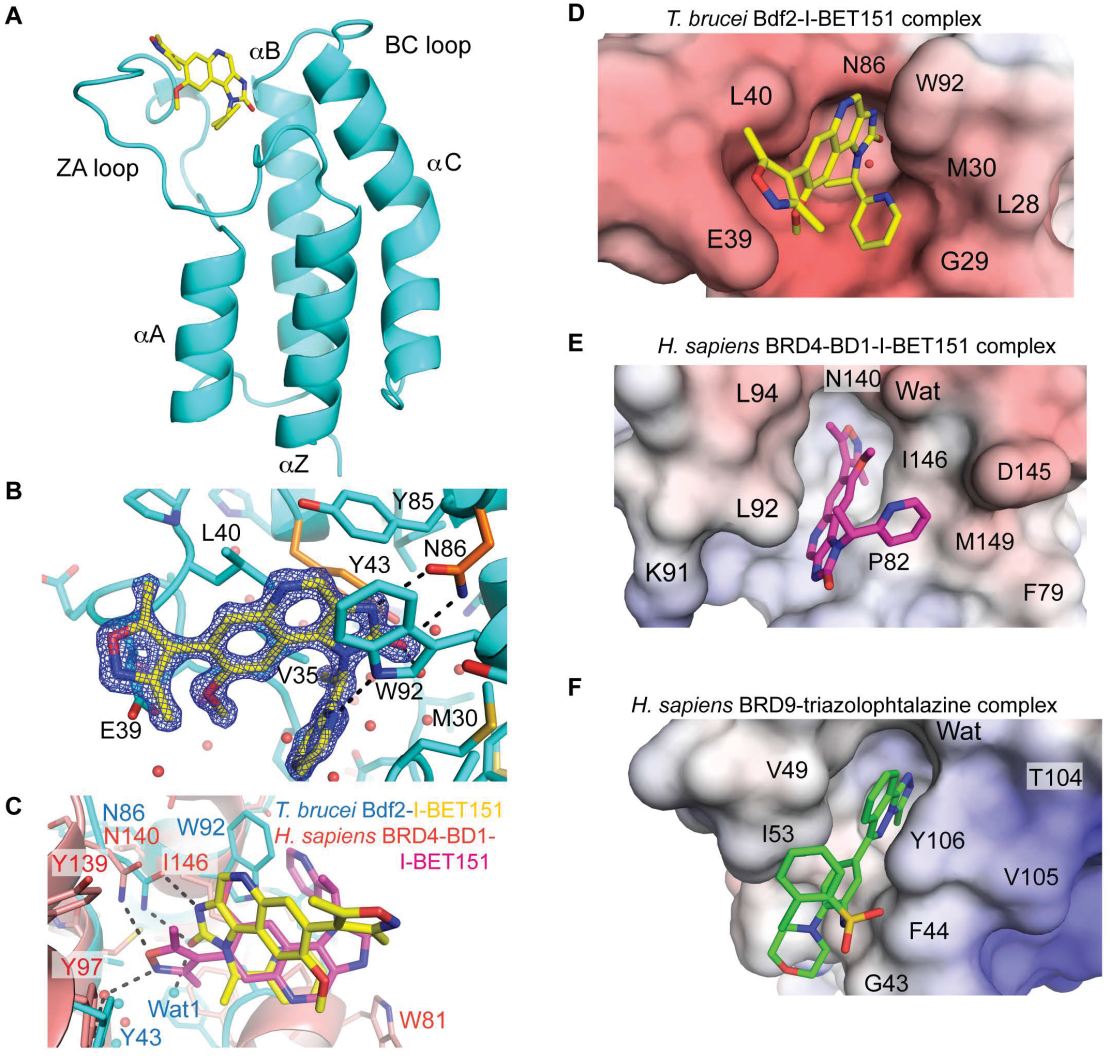
Structural analysis of I-BET151 binding to Bdf2. (A) Structure of the Bdf2 bromodomain in complex with I-BET151 (PDB code 4PKL). (B) Electron density of I-BET151 and key residues for ligand recognition. The two conserved residues mutated to alanine in the Bdf2 mutant are colored in orange. (C) Superimposition of Bdf2 (cyan) in complex with I-BET151 (yellow) onto human BRD4-BD1 (light red, 3ZYU) in complex with I-BET151 (magenta). (D to F) Electrostatic surface potential shown between -10 kT/e (red) and +10kT/e (blue) of (D) trypanosome Bdf2 bromodomain in complex with I-BET151, (E) human BRD4-BD1 in complex with I-BET151 (3ZYU), and (F) human BRD9 in complex with a triazolophtalazine inhibitor (4NQN).

To provide a blueprint towards designing high-affinity trypanosome-specific bromodomain inhibitors, we determined the crystal structure of the Bdf2 bromodomain in complex with I-BET151 at 1.25Å resolution. The overall structure adopts the canonical bromodomain fold comprising four α-helices linked by variable loop regions that form the ligand binding site [39,40] (Fig 7A and 7B). Unexpectedly, but consistent with the reduced affinity, the ligand is flipped by roughly 180° with respect to the human BRD4-BD1-I-BET151 complex and cannot adopt its classical binding mode (Fig 7C) [23]. In human BRD4-BD1, the dimethylisoxazole group mimics AcK, recognizing the conserved Asn (Asn140) and the conserved water network. In Bdf2, the dimethylisoxazole ring is solvent-exposed, while recognition of the conserved Asn takes place through a bidentate hydrogen bond to the imidazolone moiety. The new orientation is probably due to a clash of the pendent pyridine ring with the large trypanosome gatekeeper residue Trp92, which dominates ligand recognition through parallel π–π stacking and which is not found in any of the 61 human bromodomains at this position [41]. The flipped binding mode is further enforced by the narrow AcK binding slot that cannot accommodate the energetically favorable twist of the dimethylisoxazole with respect to the quinoline ring (Fig 7D). Notably, the pyridine moiety forces the imidazolone to sit high up in the AcK binding pocket, resulting in an imperfect fit with a gap at the bottom of the pocket that is filled by an additional, unconserved water molecule (Wat1, Fig 7C). By contrast, the smaller gatekeeper residue Ile146 in human BRD4-BD1 allows the twisted dimethylisoxazole to engage in the classical AcK recognition (Fig 7E).

BRD9 is one of the closest human structural homologs to Bdf2 and also possesses an aromatic gatekeeper residue (Tyr106) that recapitulates the parallel π–π stacking interaction with a triazolophtalazine compound within a narrow slot (Fig 7F). However, the negative electrostatic surface potential of Bdf2 markedly differs from the slightly positively charged BRD9 pocket, highlighting the distinct character of the Bdf2 AcK binding site, a prerequisite for developing a trypanosome-specific drug. Thus, the structure illuminates how the compound may be optimized towards selective treatments for trypanosomiasis.

## Discussion

*T*. *brucei* differentiation from the BF to the PF requires extensive reprogramming that results in drastic changes in morphology, metabolism, and protein expression. These changes are reflected at the level of gene expression, with recent studies indicating that 25%–40% of all genes are differentially expressed as the cells transition from one form to another [10,12,42]. A number of mechanisms have been shown to be involved in the developmental regulation of gene expression, including alternative splicing [42] and mRNA stability mediated by regulatory sequences within 5’ and 3’ UTRs [6–8,43]. These regulatory sequences are, in turn, bound by RNA binding proteins. For example, ZFP3 associates with EP1 and GPEET mRNA and can interact with translation factors [44,45]. Our studies indicate that there may be an additional layer of developmental regulation of gene expression that is mediated at the DNA level. We show that inhibition of bromodomain proteins cause some transcriptional changes that are similar to those observed during the physiological transition from the BF to the PF. These transcription changes have functional consequences, such as the appearance of EP1 on the cell surface and the gradual disappearance of surface VSG.

An alternate explanation for the data is that treatment with I-BET151 and/or knockdown of Bdf genes results in a global transcriptional dysregulation, such that all formerly silenced genes show increased transcription and active genes show decreased transcription. To try and determine whether this was the case for our I-BET151 time course, we split genes into those that had previously been shown to change during differentiation as defined by the Queiroz study, and those that have not. If global dysregulation were occurring, we would expect that in the group of genes not associated with differentiation, there should be an increase in median RPKM for genes that are poorly expressed at time 0, and a decrease in median RPKM for genes that are highly expressed at that time point. Genes in the “procyclic” group, as defined by Queiroz et al. are poorly expressed in BF cells. We examined the range of starting RPKMs for this group of genes and extracted genes of similar starting RPKM from the set of genes that have not previously been shown to be involved in differentiation. We then plotted the median level of gene expression over time for this group of poorly expressed genes. We did not observe a significant increase in the median RPKM for this set of genes over the course of I-BET151 treatment (S15 Fig), indicating that global disregulation is not occurring. We obtained similar results for the set of genes with high starting RPKM, whose range was chosen based on the range of expression for the genes found in the “glycolysis 1” group of genes defined by Queiroz et al. (S15 Fig). We did not observe a general decrease in median RPKM for the set of highly expressed genes that were not previously shown to be involved in differentiation over the course of I-BET151 treatment. While we have not formally disproved global disregulation, we believe these findings argue for a more specific effect of I-BET151 treatment on the set of genes known to be involved in differentiation.

In *T*. *brucei*, there is a paucity of sequence-specific DNA regulatory sequences that can serve as transcription factor binding sites. Only one transcription factor has been well characterized [46], and it appears to primarily mediate Pol I transcription. To our knowledge, enhancer-like sequences have not been identified. It is thus unlikely that transcription factors are the means by which developmental transcriptional programs are put in place. This leaves epigenetic marks and the proteins that read them as a potential mechanism for regulating developmental changes in gene expression at the DNA level. Our work implies that bromodomain protein localization could change during differentiation from the BF to the PF in the normal course of the trypanosome lifecycle, and that this may be part of the mechanism by which a new, PF-specific transcriptional program is initiated. While we have demonstrated that bromodomain proteins bind chromatin, and that binding is altered following their inhibition, technical considerations currently preclude a direct demonstration that epigenetic changes (e.g., changes in histone marks) that fundamentally change the structure of the chromatin surrounding bromodomain-bound sites are occurring. Future studies may be able to address whether changes in the patterns of histone tail modification or DNA methylation result from altered localization of chromatin readers during differentiation.

We were able to demonstrate that bromodomain protein-mediated changes in transcriptional program are reversible (Fig 4), and we theorize that this plasticity may be a significant advantage to an organism that must reprogram itself quickly to adapt to the changing environments encountered between the insect and the mammalian host. Our results are in good agreement with a very recent study in mice, where small-molecule bromodomain protein inhibition in embryonic stem cells resulted in loss of the undifferentiated state and in spontaneous onset of differentiation [16]. The surprising parallels between bromodomain-mediated maintenance of cell fate in mouse embryonic stem cells and BF trypanosomes have interesting implications for how these processes may have evolved across diverse biological systems.

We found that inhibition of bromodomain proteins caused growth arrest, both in our genetic knockdowns of Bdf2 and Bdf3 and upon treatment with I-BET151 (Fig 6 and S8 Fig), albeit with different timing in each case. This is interesting in light of the fact that long slender BF trypanosomes cease to divide as they progress to the stumpy forms that are eventually ingested by the tsetse. Transcriptional profiling has revealed that many of the gene expression changes that take place during the transition from the BF to the PF are already in motion during the stumpy stage [10,11,42], and thus some of the transcription changes that we observe in I-BET151-treated cells might be shared with cells transitioning from the long slender to the stumpy form. That is to say that it is possible that the cell cycle arrest observed upon bromodomain protein inhibition is a result of the transcriptional program that is set forth following inhibition. The Lister427 strain used in this study is not pleomorphic and thus cannot be induced to form stumpies. However, it may be that bromodomain inhibition causes the cells to acquire some characteristics of stumpy forms. That said, we were unable to detect PAD1 on the surface of I-BET151-treated cells [47], and thus do not think the drug is inducing formation of a true stumpy form. Conversely, it is possible that the growth arrest is causing transcriptional changes consistent with progression to the next lifecycle stage. However, the fact that we see an up-regulation of EP1 mRNA at 3 h following I-BET151 treatment, and that we do not observe a pronounced growth arrest in the first 24 h of treatment does not support this notion. In future studies, it will be interesting to see what effect I-BET151 has on pleomorphic strains, and whether it is able to instigate transition to the stumpy form.

We initially chose to focus on the BF cell fate because its loss could have important therapeutic implications. For example, disrupting immune evasion mechanisms specific to BF cells (e.g., antigenic variation or antibody internalization) could lend the host immune system a much-needed boost in combating infection. However, the question as to whether bromodomain proteins are important for maintaining cell fate in other lifecycle stages, such as in PF cells, is an important one to be addressed in future studies.

Evidence has accumulated over the years that chromatin structure and chromatin-interacting proteins are critical for maintaining monoallelic expression of *VSG* genes [17,48,49]. Consistent with these results, inhibiting bromodomain proteins that read AcK marks on the histone tails affects monoallelic expression. One previous study demonstrated that deletion of a histone methyltransferase caused cell cycle arrest and cell death after induction of differentiation from the BF to the PF [50]. Because many PF-specific genes were up-regulated following induction of differentiation, the authors suggested that the phenotype could be a byproduct of a cell cycle checkpoint defect, rather than a problem with initiation of differentiation. To our knowledge, our study is the first report of a chromatin factor being required to prevent premature differentiation prior to environmental cues. This is particularly interesting in light of the fact that previous reports have linked gene expression within the ESs to differentiation processes [27,29], and we hypothesize that bromodomain proteins may be one of the factors that mechanistically tie these two processes together. One scenario for how this might occur is that an unidentified signaling event could trigger removal of bromodomain proteins from the ESs, resulting in up-regulation of mRNA from many different ESs, which in turn could cause active ES attenuation and initiate a PF transcription program, as envisioned by Batram et al. Conversely, a global removal of bromodomain proteins from transcription initiation sites could be the event by which a PF-specific transcription program is initiated, with active ES attenuation and transient derepression being part of this more global differentiation program. Our observation that inhibiting the more ES-specific factor Bdf2 caused less of a change in the global transcriptome, but that Bdf2-depleted cells were still “primed” to undergo differentiation to a larger degree than wildtype cells (Fig 5E) supports the first scenario.

Our insights into this differentiation process have fundamental implications for the design of new strategies to treat trypanosomiasis, as inhibiting bromodomain proteins decreases virulence in a mouse model (Fig 6). This finding is important because trypanosomiasis is fatal if untreated and there is a paucity of drugs available for this disease [33]. Novel drug targets are necessary; as late-stage treatments are highly toxic, few new drugs are in development, and drug resistance is increasing [22]. The notion that bromodomain inhibitors can “trick” a BF trypanosome into prematurely initiating differentiation to the insect stage while still in the bloodstream imparts this class of molecules with substantial therapeutic potential. In addition to the adverse effects of I-BET151 on the parasite’s fitness itself (Fig 6A and 6B and S8 Fig), the host immune system would have specific advantages over *T*. *brucei*: (1) expression of invariant insect stage proteins on the *T*. *brucei* surface provides a “handle” for a single antibody type to recognize the parasite (in contrast to the constantly antigenically varying VSG coat that is native to *T*. *brucei* in the bloodstream) and (2) blockade of rapid antibody internalization, another hallmark of the *T*.*brucei* BF, would allow more time for host effector cells to recognize and eliminate the parasites.

While I-BET151’s low affinity for trypanosome bromodomain proteins currently limits its use in therapeutic intervention, our crystal structure of Bdf2 in complex with I-BET151 provides critical information to guide the design of a highly specific trypanosome bromodomain inhibitor. The fact that the architecture of the AcK recognition site of the Bdf2 bromodomain markedly differs from its mammalian counterparts (Fig 7) implies that a trypanosome-specific bromodomain inhibitor can in principle be developed. This is particularly relevant, because various bromodomain inhibitors such as I-BET151 affect the mammalian immune response [22], which could pose problems during an infection. Our structure provides specific clues, for example deletion of the pendent pyridine ring or expansion of the tricyclic aromatic ring system to fill the gap at the bottom of the binding site, for augmenting the drug-Bdf2 interactions to yield a trypanosome-specific, high-affinity ligand, while concurrently decreasing affinity for mammalian bromodomain proteins (Fig 7C). This would avoid compromising the immune response in the host, while inhibiting trypanosome growth, offering a promising new avenue toward therapeutic intervention for trypanosomiasis.

## Materials and Methods

### Culture Methods and Strains

*T*. *brucei* BF cells (strain Lister 427 antigenic type MITat1.2 clone 221a) were cultured in HMI-9 at 37°C. We used either a “single marker” (SM) line expressing T7 RNA polymerase and Tet repressor (TETR) [51], or a dual BES marked line (L224) that expresses VSG3 from BES7 (with *Neo* ^*R*^ downstream of the promoter) and a *Puro* ^*R*^ gene downstream of the BES2 promoter (expressing VSG2) [28]. Stable clones were maintained in HMI-9 medium containing necessary antibiotics.

### Strain Construction

Trypanosomes were maintained at the following drug concentrations, unless otherwise stated: 2.5 μg/ml, G418 (Sigma); 5 μg/ml, blasticidin (Invivogen); 5 μg/ml, hygromycin (Invivogen); 0.1 μg/ml, puromycin (Invivogen); 1μg/ml, phleomycin (Invivogen). The Bdf2^KO^ strain was generated by cloning the coding sequence of *Bdf2* (Tb427.10.7420) upstream of an HA tag in the pMOTAG5H loxP plasmid. A portion of the 3’UTR was cloned downstream of a phleomycin resistance gene and cells were selected in phleomycin following transfection with an Amaxa nucleofector kit. The remaining endogenous allele of Tb427.10.7420 was replaced with a hygromycin resistance gene using 5’ and 3’ UTR homology arms cloned into the pyrFEKO-HYGRO loxP plasmid. The pLEW100cre-EP1-6G Phleo plasmid was integrated into the cell line by selecting for Phleomycin resistance following transfection to allow for inducible CRE expression as in [52]. The Bdf3^kd^ strain was generated by cloning the coding sequence of *Bdf3* (Tb427.01.1830) upstream of an HA tag in the pMOTAG5H plasmid. A portion of the coding sequence of Tb427.01.1830 was cloned into the RNAi vector P2T7TABlue and integrated into the rDNA locus. All cell lines were checked for proper integration using PCR. The cell line used for ChIP (HSTB-907) was generated by first randomly integrating a cassette containing puromycin-resistance gene, luciferase, and EmGFP (pHJ1, reference) at a silent BES promoter, and then targeting a blasticidin resistance marker at the active BES promoter (BES1-VSG2). To determine which silent BES promoter was targeted with pHJ1, in situ switched cells were selected at 100x higher concentration of puromycin (10 μg/ml) and confirmed by GFP-FACS (HSTB-717). VSG RNA analysis confirmed that the switched cells express VSG11 in BES11.

### I-BET151 and Dox Treatment

Cells were treated with 20 μM I-BET151 in DMSO to inhibit bromodomains, or with 1 μg/ml doxycyclin to induce deletion of *Bdf2* or RNAi against *Bdf3*.

### Western Blotting

5–10 million cells were suspended in 2X Laemmli Sample Buffer (Biorad 161–0737) and separated on a 4%–20% SDS gradient gel. After transfer to PVDF membrane, blots were blocked in 5% milk in PBS and probed with anti-HA HRP (Miltenyi 130-091-972), anti-H3 (Abcam 1791), or anti-tubulin (gift from Keith Gull) followed by anti-rabbit IgG-HRP or anti-mouse IgG-HRP.

### Flow Cytometry

1–2 million treated and control cells were suspended in HMI-9 and stained with fluorescence conjugated anti-VSG2 or anti-VSG3 for 10min with vortexing [53]. Cells were washed twice in HMI-9 before analysis on either a BD LSRII or a BD FACSCalibur. PI (BD 51–66211) was added to the final suspension for dead cell exclusion at 0.75μg/ml.

### Differentiation Experiments

L224, Bdf2^KO^, or Bdf3^kd^ trypanosomes were treated for 23 d with I-BET151 and Dox, respectively. Cells were washed twice in media to eliminate I-BET151 or Dox, resuspended in HMI-9 and subjected to 24 h of treatment at 27°C, or in 6mM cis-aconitate (Sigma A3412). Control cells were kept at 37°C. Cells were stained with anti-EP1 (Cedarlane CLP001A) and an anti-mouse IgG FITC-labeled secondary antibody (BD 349031) and analyzed by flow cytometry.

### Antibody Internalization

L224, Bdf2^KO^, or Bdf3^kd^ trypanosomes were treated for 2–3 d with I-BET151 and Dox, respectively. Cells were stained with unconjugated anti-VSG3 (L224) or anti-VSG2 (Bdf2^KO^ or Bdf3^kd^) primary antibody and washed. Cells were subjected to a 5 min incubation at either 4°C (controls) or at 37°C to allow internalization to take place. Cells were immediately fixed in 1% formaldehyde and stained with a FITC-conjugated secondary antibody (BD 349031) to measure the remaining amount of primary antibody on the surface. Cells were then analyzed by flow cytometry. For immunofluorescence, cells were adhered to poly-L-lysine coverslips following fixation, blocked in PBS with 0.2% cold water fish gelatin (Sigma G7765) and 0.5% (w/v) BSA and stained with anti-mouse IgG FITC (BD 349031) at 1:1,000 for 1 h. Cells were washed, counterstained with DAPI and imaged on a DeltaVision Image Restoration inverted Olympus IX-70 microscope (Applied Precision).

### Movies

L224 trypanosomes were treated with I-BET151 or DMSO for 2–3 d and 1.5 million live cells in media were imaged in a MatTek glass-bottom dish (P35G-0.17-14-C) at 1 ms intervals on a DeltaVision Image Restoration inverted Olympus IX-70 microscope (Applied Precision).

### Cell Cycle PI Staining

Cells were fixed for 5 min in 1% formaldehyde in PBS. After 3 washes, cells were resuspended in solution containing 0.2 mg/ml RNAse and 0.05 mg/ml PI in PBS. Cells were incubated for 2 h at 37°C and analyzed by flow cytometry.

### Growth

Trypanosomes treated with either I-BET151 or Dox were diluted to 100,000 cells/ml in HMI-9 every 24 h, counted on a hemacytometer and compared to DMSO control-treated cells or untreated cells (for Dox treatments).

### Viability

Three independent cultures of trypanosomes were treated with I-BET151 for 3 d or with DMSO as a control. Following treatment cells were washed, counted on a hemacytometer and then serially diluted to a concentration of 5 cells/100 μL HMI9 or 50 cells/10 0μL HMI9 with no drug. They were plated on three 96-well plates and cultured for 3–4 d at 37°C before quantifying the number of wells growing in each plate. An aliquot of each culture was subjected to PI staining (BD 51–66211) at 0.75 μg/ml to assess viability and countbright beads (Life technologies C36950) were added to quantify cell growth. Washed cells were allowed to recover for 3 d before being subjected to PI staining again.

### RNA-Seq

RNA was extracted from treated or control cells using RNA Stat-60 (Tel-Test) following the manufacturer’s protocol and quantified on a NanoDrop2000c. Following DNAse treatment on 5 μg of RNA, poly(A)^+^ sequencing libraries were prepared using the NEBNext Poly(A) mRNA Magnetic Isolation Module (E7490S) followed by the NEBNext Ultra Directional RNA library prep kit for Illumina (E7420L) or by the Rockefeller University Genome Sequencing Center using the Illumina kit. Sequencing was performed on an Illumina HiSeq 2000 sequencer using 100 bp reads. Reads were trimmed for quality with TrimGalore from Babraham Bioinformatics (http://www.bioinformatics.babraham.ac.uk/projects/trim_galore/) using stringency setting three and aligned first to the reference genome (Tb927v5.1) and then to the Lister 427 VSGnome [1] using bowtie [54] to uniquely align reads and allowing for two mismatches. For the ESAG analysis, reads were uniquely aligned allowing for zero mismatches to unambiguously determine the BES. Example commands are given below.

trim_galore—stringency 3 L224-1_CGATGT_L002_R1_001.fastq.gz

bowtie—best—strata -t -v 2 -a -m 1—sam—un UN_L224-iB_1_140804_tb927_v5.1 /Users/dschulz/To_Clean/bowtie-0.12.8/indexes/140804_tb927_v5.1 L224-1_CGATGT_L002_R1_001.fq L224-iB_1_140804_tb927_v5.1.sam

bowtie—best—strata -t -v 0 -a -m 1—sam /Users/dschulz/To_Clean/bowtie-0.12.8/indexes/141107_VSGs_CDSs_ESAGs UN_L224-iB_1_140804_tb927_v5.1 L224-iB_1_141107_VSGs_CDSs_ESAGs_m1_v0.sam

RPKM values were quantified using SeqMonk from Babraham Bioinformatics (http://www.bioinformatics.babraham.ac.uk/projects/seqmonk) or, in the case of *Vsg*- and *ESAG*-aligned reads, custom python scripts. All sequencing reads were performed in biological duplicate or triplicate and RPKM values were averaged. For reads aligning to the genome, DESeq [24] was used to generate *p*-adjusted values using the negative binomial test for differences between the base means for two conditions with the following command: nbinomTest(cds, condA, condB). For reads aligning to *VSG*s and *ESAG*s, Q values were calculated using SeqMonk’s statistical replicate set test, which adjusts the *p*-values using Benjamimi and Hochberg correction. Note that the Bdf2 dataset had to be sequenced more deeply for *ESAG* analysis because of the stringent parameters used (unique alignments with no mismatches). This dataset was sequenced in duplicate rather than triplicate, so no *p*-values were generated for the Bdf2 *ESAG* analysis shown in S9 Fig. Plots from RNA-seq were generated using python’s matplotlib library. Boxplots were generated using the command plt.boxplot(data). Scatterplots were generated using plt.scatter(x,y). All other plots were generated using plt.plot(data).

### GSEA

GSEA analysis [25] was conducted using software available at http://www.broadinstitute.org/gsea/index.jsp. We used custom sets of functional groups and hierarchical clusters defined in [35]. These sets are provided as Additional File 3 and Additional File 4 in S1 Data. For time course data, GSEA was run using the Pearson metric for ranking genes in a time series with the following parameters:

Permutations: 1000

Collapse dataset to gene symbols: False

Permuation type: gene_set

Enrichment statistic: weighted

Metric for ranking genes: Pearson

Gene list sorting mode: real

Gene list ordering mode: descending

Max size: 500

Min size: 1

An example command is given below

java -Xmx512m xtools.gsea.Gsea -res /Users/danaeschulz/Science/Tryp_Science/15-7-7_L224_iBET_RNAseq_timecourse/15-7-19_L224_timecourse_Gsea_analysis/L224_timecourse_continuous_data_for_GSEA.txt -cls /Users/danaeschulz/Science/Tryp_Science/15-7-7_L224_iBET_RNAseq_timecourse/15-7-19_L224_timecourse_Gsea_analysis/L224_timecourse_continuous_profile.cls#IncreasingProfle -gmx /Users/danaeschulz/Science/Tryp_Science/15-7-19_GSEA_analysis_L224_iBET_Bdf3_Bdf2/mappable_clayton_clusters_GMT.gmt -collapse false -mode Max_probe -norm meandiv -nperm 1000 -permute gene_set -rnd_type no_balance -scoring_scheme weighted -rpt_label my_analysis -metric Pearson -sort real -order descending -include_only_symbols true -make_sets true -median false -num 100 -plot_top_x 20 -rnd_seed timestamp -save_rnd_lists false -set_max 500 -set_min 1 -zip_report false -out /Users/danaeschulz/gsea_home/output/aug06 -gui false

For analysis at single time points, genes were ranked using Signal2Noise with the following parameters:

Permutations: 1,000

Collapse dataset to gene symbols: False

Permuation type: gene_set

Enrichment statistic: weighted

Metric for ranking genes: Signal2Noise

Gene list sorting mode: real

Gene list ordering mode: descending

Max size: 500

Min size: 1

An example command is given below

java -Xmx512m xtools.gsea.Gsea -res /Users/danaeschulz/Science/Tryp_Science/15-7-7_L224_iBET_RNAseq_timecourse/15-7-19_L224_timecourse_Gsea_analysis/L224_timecourse_continuous_data_for_GSEA.txt -cls /Users/danaeschulz/Science/Tryp_Science/15-7-7_L224_iBET_RNAseq_timecourse/15-7-19_L224_timecourse_Gsea_analysis/L224_timecourse_continuous_profile.cls#IncreasingProfle -gmx /Users/danaeschulz/Science/Tryp_Science/15-7-19_GSEA_analysis_L224_iBET_Bdf3_Bdf2/mappable_clayton_clusters_GMT.gmt -collapse false -mode Max_probe -norm meandiv -nperm 1000 -permute gene_set -rnd_type no_balance -scoring_scheme weighted -rpt_label my_analysis -metric Signal2Noise -sort real -order descending -include_only_symbols true -make_sets true -median false -num 100 -plot_top_x 20 -rnd_seed timestamp -save_rnd_lists false -set_max 500 -set_min 1 -zip_report false -out /Users/danaeschulz/gsea_home/output/aug06 -gui false

### ChIP and ChIP-Seq

Chromatin IPs were performed as in [35] except that a rabbit anti-HA antibody was used (Sigma H6908), and DNA was purified by phenol-chloroform extraction or with Ampure beads (Agencourt A63880).

ChIP qPCR primers are listed in S13 Table. ChIP-Seq libraries were made as in [35] and run on an Illumina HiSeq 2000 sequencer using 50 bp reads. Reads were trimmed for quality with TrimGalore and aligned to the reference genome (Tb927v5) using bowtie [54] to uniquely align reads and allowing for two mismatches. The MACS algorithm was used to identify peaks in the ChIP-seq data set [55]. An example command is as follows macs14 -t A5IP_tb927v5fix5with12.bed -c A5In_tb927v5fix5with12.bed -n A5IP_MACS -f BED -g 23650671. Each replicate was compared to input to obtain an initial set of called peaks. Bedtools was then used to compare the two replicates for each Bdf, and return only those peaks that were called in both replicates. Finally, peaks called by MACS in the untagged control were eliminated from the final set of called peaks. For Fig 5B, only peaks with an FDR of <0.1 were used for the analysis. All sequencing reads were performed in biological duplicate or triplicate.

### Q-PCR

For Q-PCR to quantify transcription, RNA was extracted from treated or control cells using RNA Stat-60 (Tel-Test) following the manufacturer’s protocol and quantified on a NanoDrop2000c. 5 μg of RNA was used to generate cDNA using random hexamer primed Superscipt III Reverse transcriptase (Life Technologies 18080093) according to the manufacturer’s protocol. For Q-PCR to quantify ChIP, DNA was purified using Agencourt Ampure XP beads (Agencourt A63880). For amplification, cDNA or ChIP purified DNA was amplified using 2X Sybr green master mix (Life Technologies4309155) and primers and quantified on an Applied Biosystems 7900HT Sequence Detection System. Primers used for Q-PCR are listed in S13 Table.

### Imagestream

I-BET151-treated trypanosomes and control cells were stained with antibodies against *VSG* in the transcriptionally active ES (VSG3) and a *VSG* in a silent ES (VSG2 or VSG13) as well as DAPI for dead cell exclusion. Cells were analyzed and photographed on an Amnis ImageStream-X flow cytometer.

### Trypanosome Infections In Vivo

C57/B6 or *RAG-/-* (Jackson Labs #002216) mice were fed with Dox feed (Doxycycline 5053 200 ppm) or regular feed (Lab Diet 5053) for 24–48 h before infection or following the appearance of parasites. One million Bdf2^KO^ or Bdf3^kd^ trypanosomes suspended in HMI-9 were IP injected into each mouse. Mice were kept on either Dox or regular feed for the remainder of the experiment. Parasitemia was monitored daily starting 48 h postinfection by tail bleed, dilution, and counting on a hemacytometer. Mice were monitored for 7–20 d. Control experiments were performed where parasitemia was monitored in mice infected with wildtype parasites fed on either Dox feed or regular feed to ensure that Dox did not influence virulence (control_mouse_experiment file in S6 Data). Infections proceeded similarly in these two groups. For the I-BET151 experiments, trypanosomes were pretreated in vitro for 2–3 d with 20 μM, and 50 trypanosomes were injected by IP into each mouse. Mice were infected between 4–8 wk of age. All studies were conducted in accordance with the GSK Policy on the Care, Welfare and Treatment of Laboratory Animals and were reviewed by the Institutional Animal Care and Use Committee at GSK and by the ethical review process at The Rockefeller University.

### Protein Expression, Purification, Crystallization, Data Collection, Structure Determination, and Refinement

DNA fragments of wild-type Bdf2^9-123^ and wild-type Bdf3^32-150^ were amplified by PCR from genomic DNA, cloned into a pET28a vector (Novagen) containing an N-terminal PreScission protease (GE Healthcare) cleavable His_6_-tag, and overexpressed in *E*. *coli*. Mutations in Bdf2^9-123^ (Y43A and N86A) and Bdf3^32-150^ (Y80A and N124A) were introduced by overlap extension PCR mutagenesis. For formation of the inhibitor complex, 10 mg/ml of purified Bdf2^9-123^ was mixed in a 1:1 molar ratio with I-BET151 and incubated for 1 h on ice. The crystallization solution consisted of 2.2 M Na/K PO_4_, pH 6.6, 0.1 M NaOAc pH 4.1, and 4% (v/v) 1-propanol. Crystals grew within two weeks. X-ray diffraction data were collected at the X29 beamline at the National Synchrotron Light Source (NSLS) at the Brookhaven National Laboratory (BNL). Diffraction data were processed in HKL2000 [56], the structure was solved by molecular replacement using individual α-helices one at a time as search templates with the program Phaser [57], and an initial core model was built by ARP/warp [58]. Model building was performed in O [59] and Coot [60]. The model of Bdf2 was verified by peaks in an anomalous difference density map from data collected at 1.73Å that coincided with the sulfur positions of the cysteine and methionine residues. The final model spanning residues 9–114 was refined in Phenix [61] to an R_free_ of 17.9% with excellent stereochemistry as assessed by MolProbity [62]. Details for data collection and refinement statistics are summarized in S11 Table. Atomic coordinates and structure factors have been deposited with the Protein Data Bank under PDB code 4PKL.

### Isothermal Titration Calorimetry

ITC measurements were performed at 15°C using a MicroCal auto-iTC200 calorimeter (MicroCal, LLC). Protein samples were extensively dialyzed against a buffer containing 20 mM Hepes, pH 7.5, 150 mM NaCl, 0.5 mM TCEP, and 1% DMSO. Typically 5 μL of 2.0 mM protein was injected into 0.4 mL of 0.2 mM ligand in the chamber every 150 s. Baseline-corrected data were analyzed with ORIGIN software.

### Anti-VSG2 and Anti-VSG3 Antibodies

Antibodies were generated at the Memorial Sloan Kettering Cancer Center Monoclonal Antibody Facility and conjugated with either A488 or APC fluorophores.

### Statistical Tests

All statistical tests were Student’s *t* tests except for those performed in Fig 6C and 6D. In Fig 6C and 6D, log-rank Mantel-Cox tests were performed on survival of the three groups. For Fig 6E, because parasites were below the lower level of quantification (LLQ), we assigned values = ½(LLQ) to those mice for which we could not get quantifiable parasite counts. A Mann-Whitney U test was then performed to compare Dox-feed and regular-feed groups of mice. The LLQ for these experiments is 5.56 x 10^5^ parasites/ml.

## Supporting Information

S1 Data Numerical data for Fig 1. (ZIP)

S2 Data Numerical data for Fig 2. (ZIP)

S3 Data Numerical data for Fig 3. (ZIP)

S4 Data Numerical data for Fig 4. (ZIP)

S5 Data Numerical data for Fig 5. (ZIP)

S6 Data Numerical data for Figs 5 and 6. (ZIP)

S7 Data Numerical data for S1 Fig. (ZIP)

S8 Data Numerical data for S2 Fig. (ZIP)

S9 Data Numerical data for S3 Fig. (ZIP)

S10 Data Numerical data for S4 Fig. (ZIP)

S11 Data Numerical data for S5 Fig. (ZIP)

S12 Data Numerical data for S6 Fig. (ZIP)

S13 Data Numerical data for S8 Fig. (ZIP)

S14 Data Numerical data for S9 Fig. (ZIP)

S15 Data Numerical data for S10 Fig. (ZIP)

S16 Data Numerical data for S11 Fig. (ZIP)

S17 Data Numerical data for S12 Fig. (ZIP)

S18 Data Numerical data for S13 Fig. (ZIP)

S19 Data Numerical data for S14 Fig. (ZIP)

S20 Data Numerical data for S15 Fig. (ZIP)

S1 Fig Temporal analysis of transcriptional changes induced by I-BET151 treatment for genes within functional groups. (A–D) DESeq was used to call genes with significantly altered transcription between I-BET151 treated cells and DMSO-treated control cells for every time point using a *p*-adjusted cutoff of < 0.1. For each functional group, the percentage of genes within that group that were significantly altered was calculated for each time point and plotted. All groups with a GSEA FDR of <0.1 are shown. (A–C) Middle induced functional groups. (D) Late induced functional groups. Numerical data for S1 Fig is in S7 Data.
(EPS)

S2 Fig Temporal analysis of transcriptional changes induced by I-BET151 treatment for genes within hierarchically clustered sets. (A–E) DESeq was used to call genes with significantly altered transcription between I-BET151 treated cells and DMSO-treated control cells for every time point using a P adjusted cutoff of < 0.1. For each cluster, the percentage of genes within that cluster that were significantly altered was calculated for each time point and plotted. All groups with a GSEA FDR of <0.1 are shown. See text for description of clusters. (A-D) Middle induced clusters. (E) Late induced clusters. Numerical data for S2 Fig is in S8 Data.
(EPS)

S3 Fig Transcriptional changes induced by I-BET151-treated cells mimic those induced by differentiating cells. (A) Plot showing median RPKM for all genes within the indicated clusters at each time point after induction with I-BET151 (solid lines). For comparison, median expression values for all genes within the indicated clusters were derived from data generated in [12] and plotted as dashed lines. Colors between solid and dashed lines are matched for each cluster. All clusters with a GSEA FDR of <0.1 and where >70% of genes were up-regulated or down-regulated at 48 h of I-BET151 treatment are plotted. The gray dotted line is a reference line. (B) Examples of 3 clusters that do not match our criteria of a GSEA FDR of <0.1 and where >70% of genes were up-regulated or down-regulated at 48 h. Numerical data for S3 Fig is in S9 Data.
(EPS)

S4 Fig Bromodomain proteins maintain monoallelic expression of *VSG* genes. (A) Schematic of VSG locations throughout the genome, ES, expression site; ESAG, Expression Site associated gene; P, promotor. * refers to the fact that one metacyclic VSG is located in an atypical metacyclic ES. (B) MA plot for RNA-seq experiment plotting mean RPKM values against log_2_(fold change) of I-BET151 over DMSO-treated control cells for all annotated *VSG* genes in the VSGnome. Red signifies *VSG*s with a *p*-adjusted value of < 0.1. ^ marks RPKM level for *VSG3* in the active ES. (C) Top, I-BET151-treated trypanosomes and control cells (not shown) were stained with antibodies against VSG at a transcriptionally active ES (VSG3) and a VSG at a silent ES (VSG2 and VSG13, respectively) as well as DAPI for dead cell exclusion. Cells were analyzed and photographed on an Amnis ImageStream-X flow cytometer. Two examples of double-expressing cells are shown. Bottom, flow cytometry plots for the cells prepared for imagestream showing that double VSG expressors are a small proportion of the total population. Left panels, DMSO-treated control cells. Right panels, I-BET151-treated cells. (D) Fold changes in RPKM values for *ESAG*s located within the active ES for I-BET151 treated cells compared to DMSO-treated control cells. *ESAG*s are ordered by genomic location along the ES. ES, expression site, pg, pseudogene. (E) MA plot for RNA-seq experiment plotting mean RPKM values against log_2_(fold change) of I-BET151 over DMSO-treated control cells for annotated *ESAG*s in transcriptionally silent ESs. Red signifies *ESAG*s with a *p*-adjusted value of < 0.1. Numerical data for S4 Fig is in S10 Data.
(EPS)

S5 Fig *VSG*s and *ESAG*s show altered transcription late in the I-BET151 time course. (A) The median of log_2_(RPKM) for all *ESAG* genes in silent ESs is plotted over a time course of I-BET151 treatment. (B) The median of log_2_(RPKM) for all inactive *VSG* genes is plotted over time course of I-BET151 treatment. Numerical data for S5 Fig is in S11 Data.
(EPS)

S6 Fig Isothermal titration calorimetry measuring binding of Bdf2 to (+)-JQ1 (A) and binding of Bdf3 to (+)-JQ1 (B). Numerical data for S6 Fig is in S12 Data.
(EPS)

S7 Fig Inducible strains constructed to inhibit Bdf2 or Bdf3. **(A)** Schematic of constructs used to create the Bdf2^KO^ and Bdf3^kd^ strains. Triangles, loxP sites, P, Dox-inducible promoter. cBdf3, DNA complementary to a portion of the *Bdf3* gene for RNAi induction. **(B)** Western blot of Bdf2^KO^ cells treated with Dox to delete *Bdf2*. Membranes were blotted with anti-HA antibodies; anti-H3 antibodies were used as a loading control. **(C)** Western blot of Bdf3^kd^ cells treated with Dox to inhibit Bdf3. Membranes were blotted with anti-HA antibodies; anti-H3 antibodies were used as a loading control. The construct is slightly leaky, as untreated Bdf3^kd^ cells express slightly less Bdf3 protein than parental cells.
(EPS)

S8 Fig Bromodomain inhibition causes growth defects. (A) Growth curves comparing growth of Dox-treated Bdf2^KO^ cells to untreated controls. Cells were diluted to 100,000 cells/ml and incubated at 37°C for 24 h when they were counted on a hemacytometer and diluted back to 100,000 cells/ml. This procedure was repeated for all the days indicated.* indicates a *p*-value of < .05 derived from a one-tailed Student’s T test. (B) Same as (A) except comparing Bdf3^kd^ cells to untreated controls. Top panel, first 24 h of Dox treatment, bottom panel, measurements over days instead of hours. (C) Flow cytometry of PI-stained cells to measure DNA content in Dox-treated Bdf3^kd^ cells (top) and Bdf2^KO^ cells (bottom) compared to untreated controls. (D) Western blot measuring Bdf2-HA or Bdf3-HA tagged protein levels following Dox treatment for 12, 24, or 48 h of induction. Tubulin is used as a loading control. Numerical data for S8 Fig is in S13 Data.
(EPS)

S9 Fig Genetic depletion of Bdf3, but not Bdf2, causes transcriptional changes similar to those seen in cells differentiating from the BF to the PF. (A) Left, boxplots showing log_2_(RPKM) values for all genes within functional groups previously shown to be differentially expressed in the transition from BF to PF [12]. Each group is demarcated by alternating white and gray shading and contains two boxplots representing untreated control cells (left) and Dox-treated Bdf3^kd^ cells (right). All functional groups with a GSEA FDR < 0.1 and >70% of genes up- or down-regulated within each group at 48h post-induction are shown. Right, same as left except for hierarchically clustered groups. The median for each set of genes is shown as a red line. Whiskers demarcate the inner quartile range. Outliers are shown as blue dots. Aminoacid, Amino acid synthesis or degradation, chap, Chaperone or protein-folding-related function, transporter, Transporters, channels, permeases, mitenz, mitochondrial enzymes, glyc, Glycosomal enzymes, glycBS, proteins required for high rate of glucose and glycerol metabolism in BFs, flag, flagellar genes, procyc, procyclic surface antigens, nucleotide, Base, nucleotide, nuceloside, pteridine, folate metabolism, vestrans, proteins required for intracellular protein and vesicular transport. (B) Same as A except for Bdf2^KO^ cells. Numerical data for S9 Fig is in S14 Data.
(EPS)

S10 Fig Genetic depletion of bromodomain proteins perturbs monoallelic expression of *VSG* genes. (A) Boxplots showing log_2_(RPKM) values for *VSG* genes located in expression sites, metacyclic expression sites, and minichromosomes in Dox-treated Bdf2^KO^ (left plot) or Bdf3^kd^ cells (right plot) vs. untreated cells. Within each alternating white or gray column, values for untreated cells are shown on the left, and Dox-treated cells are shown on the right. *p*-Values shown at the bottom of each white or gray column are derived from a student’s *t* test. *p < 0.05, **p < 0.01, ***p < 0.001. Note that the test was not performed on the set of all VSGs. (B) MA plot showing average RPKM against log_2_(fold change) for treated cells over untreated cells for all annotated *VSG* genes in the genome. ^ indicates the active VSG. (C) Top, Schematic of the ES reporter strain containing a blasticidin resistance gene, a VSG pseudogene (pseudo) and *VSG2* at the active site. Green fluorescent protein (*GFP*) and *VSG11* mark one of the silent ESs. A, active ES, S, silent ES. Bottom left, ChIP experiment followed by q-PCR to compare the amount of DNA in anti-HA Bdf2 pulldowns compared with untagged controls at the indicated ES regions. Bottom right, ChIP experiment followed by q-PCR to compare the amount of DNA in anti-HA Bdf2 pulldowns in DMSO or I-BET151-treated cells at the indicated ES regions. (D) Top, schematic of the active and one silent ES in the strain used for Bdf3-HA ChIP. Bottom, ChIP experiment followed by Q-PCR to compare the amount of DNA in anti-HA Bdf3 pulldowns compared with untagged controls at the indicated ES regions. Numerical data for S10 Fig is in S15 Data.
(EPS)

S11 Fig *ESAG* gene transcription increases in Bdf2^KO^ and Bdf3^kd^ cells. MA plot showing average RPKM against log_2_(fold change) for treated cells over untreated cells for all annotated inactive ES *ESAG* genes. *ESAG* genes with *p*-adjusted values of < 0.1 are shown in red. For this sequencing run Bdf2^KO^ cells were run in duplicate so no *p*-values are shown. Numerical data for S11 Fig is in S16 Data.
(EPS)

S12 Fig I-BET151 alters Bdf3 binding. (A) ChIPs were performed on Bdf3-HA tagged cells at the indicated times following treatment with I-BET151 and Q-PCR was then used to quantify the amount of DNA from anti-HA pulldowns at peaks of Bdf3 binding that had previously been identified by ChIP-seq. ESP1, expression site promoter region. URA3 is used as a negative control. (B) ChIP-seq binding tracks for the regions tested in (A). The ES promoter was not characterized by ChIP-seq due to mapping issues, and so this track is not displayed. Numerical data for S12 Fig is in S17 Data.
(EPS)

S13 Fig Transcriptional changes as a result of knockdown or knockout of Bdf proteins can be observed at 6 h following Dox treatment. (A) Q-PCR experiment to measure transcript levels using RNA isolated at the indicated time points following induction with Dox to knockdown Bdf3. Tb927.10.9400 did not show Bdf3 dependent transcriptional changes in the Bdf3 RNA-seq experiment and is used here as a negative control. (B) Same as (A) except RNA was isolated from Bdf2^KO^ cells and *VSG*s from silent ESs are tested. Tb927.10.9400 is used here as a negative control. Meta, metacyclic. Numerical data for S13 Fig is in S18 Data.
(EPS)

S14 Fig I-BET151-treated cells are viable. (A) Three independent cultures of trypanosomes treated with I-BET151 for 3 d, washed, and then plated at the indicated concentrations in 3 separate 96-well plates and compared to DMSO-treated controls. After 4 d, the percent of wells with growing trypanosomes was quantified. (B) Three independent cultures of trypanosomes treated with I-BET151 were stained with PI to determine the percent of dead cells in the population and were compared to DMSO-treated controls. Cells were washed and allowed to recover for 3 d and then stained with PI again to determine the percent of dead cells in the culture. (C) An equivalent volume of I-BET151 or DMSO-treated control cells was harvested to perform the PI stains in (B). We added countbright beads to the PI stained cells to determine the number of cells per unit volume of culture analyzed. I-BET151 treated cells did not grow as well as their DMSO-treated counterparts either pre- or post-recovery. Numerical data for S14 Fig is in S19 Data.
(EPS)

S15 Fig Treatment with I-BET151 does not cause widespread transcriptional disregulation. (Left) RNA-seq experiment showing the median expression over a timecourse of I-BET151 treatment for the group of genes who’s expression at time 0 ranges from .05–50 RPKM and that were not previously shown to be associated with differentiation. This range of RPKMs represents the range of starting expression for genes found in the “procyclic” group, which is shown for comparison by dashed lines. (Right) Same as left except a higher RPKM range was chosen, ranging from 200–3,900 RPKM, which encompasses the range of starting expression for genes involved in glycolysis, shown for comparison by dashed lines. Numerical data for S15 Fig is in S20 Data.
(EPS)

S1 Movie Movement of DMSO-treated control trypanosomes in media. (MOV)

S2 Movie Movement of 3 d I-BET151-treated trypanosomes in media. (MOV)

S1 Table GSEA enrichment and FDR values for hierarchically clustered gene sets defined in [12] using RNA-seq data for trypanosomes treated for 2 d with I-BET151 compared to controls. >70% genes up- or down-regulated shown in bold. GSEA FDR < 0.1 shown in bold.
(XLSX)

S2 Table GSEA enrichment and FDR values for gene sets organized by functional group defined in [12] using RNA-seq data for trypanosomes treated for 2 d with I-BET151 compared to controls. >70% genes up- or down-regulated shown in bold. GSEA FDR < 0.1 shown in bold.
(XLSX)

S3 Table GSEA enrichment and FDR values for gene clusters defined in [12] using RNA-seq data time series data for trypanosomes generated from treatment with I-BET151 for 3, 6, 12, 24, and 48 h. >70% genes up- or down-regulated shown in bold. GSEA FDR < 0.1 shown in bold.
(XLSX)

S4 Table GSEA enrichment and FDR values for functional groups defined in [12] using RNA-seq data time series data for trypanosomes generated from treatment with I-BET151 for 3,6,12,24, and 48h. >70% genes up- or down-regulated shown in bold. GSEA FDR < 0.1 shown in bold.
(XLSX)

S5 Table Percentage of genes within each indicated functional group called by DESeq as differentially expressed at each time point using a cutoff of *p* < 0.1. (XLSX)

S6 Table Percentage of genes within each indicated gene cluster called by DESeq as differentially expressed at each time point using a cutoff of *p* < 0.1. (XLSX)

S7 Table GSEA enrichment and FDR values for gene sets organized by functional group defined in [12] using RNA-seq data for Bdf3^kd^ trypanosomes treated with Dox compared to controls. >70% genes up- or down-regulated shown in bold. GSEA FDR < 0.1 shown in bold.
(XLSX)

S8 Table GSEA enrichment and FDR values for gene clusters defined in [12] using RNA-seq data for Bdf3^kd^ trypanosomes treated with Dox compared to controls. >70% genes up- or down-regulated shown in bold. GSEA FDR < 0.1 shown in bold.
(XLSX)

S9 Table GSEA enrichment and FDR values for functional groups defined in [12] using RNA-seq data for Bdf2^KO^ trypanosomes treated with Dox compared to controls. >70% genes up- or down-regulated shown in bold. GSEA FDR < 0.1 shown in bold.
(XLSX)

S10 Table GSEA enrichment and FDR values for gene clusters defined in [12] using RNA-seq data for Bdf2^KO^ trypanosomes treated with Dox compared to controls. >70% genes up- or down-regulated shown in bold. GSEA FDR < 0.1 shown in bold.
(XLSX)

S11 Table Data collection and refinement statistics. (DOCX)

S12 Table List of genes categorized by both functional group and cluster. (XLSX)

S13 Table Primers used for Q-PCR. (XLSX)

S14 Table Accession numbers for RNA-seq. (XLSX)

## Abbreviations

AcK: acetyl-lysine
BET: bromodomain and extraterminal domain
BES: bloodstream expression site
BF: bloodstream form
DMSO: Dimethyl sulfoxide
Dox: Doxycyclin
ES: expression site
*ESAG*: *Expression Site Associated Gene*
FDR: false discovery rate
GPI: glycosyl phosphatidylinositol
GSEA: Gene Set Enrichment Analysis
ISG: invariant surface glycoproteins
MACS: Model-based Analysis for ChIP-Seq
PF: procyclic form
PI: propidium iodide
PTU: polycistronic transcription units
RPKM: reads per kilobase per million mapped reads
TSS: Transcription Start Site
TTS: Transcription Termination Site
VSG: Variant Surface Glycoprotein
