# Supplementary figures and images for: Bromodomain Proteins Contribute to Maintenance of Bloodstream Form Stage Identity in the African Trypanosome
Source: PLoS Biol. 2015 Dec 8;13(12):e1002316. doi: 10.1371/journal.pbio.1002316 (PMC4672894; doi:10.1371/journal.pbio.1002316)

# 1B

Forward/Side Scatter

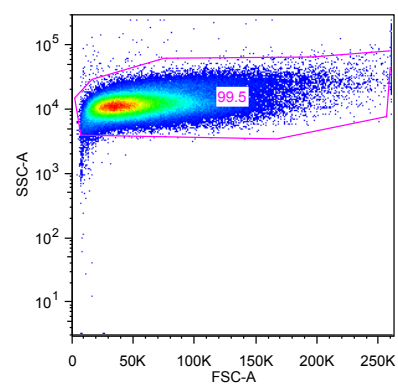

DAPI neg

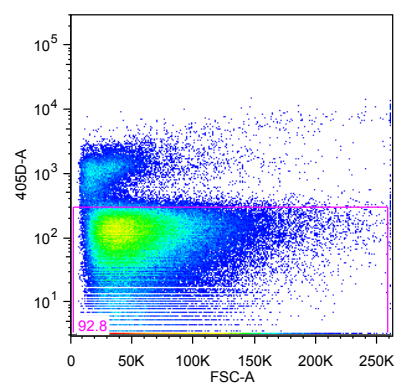

Final

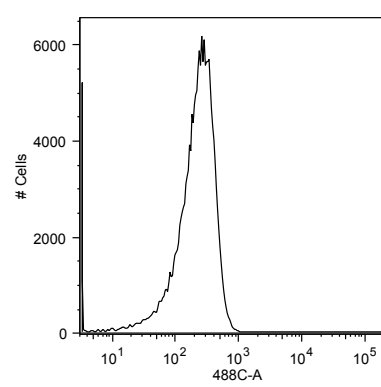

Supplement: S1 Data — (ZIP) [file pbio.1002316.s001.zip › S1_Data/FACS_Data_Fig_1B/Fig_1B_Gating.pdf]

# 3A

Forward/Side Scatter

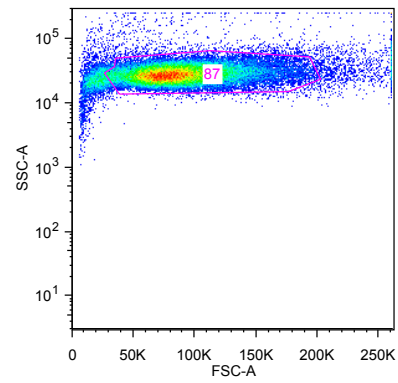

Final

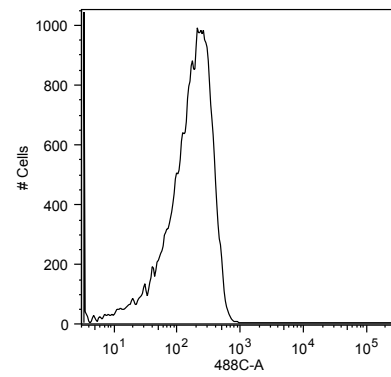

Supplement: S3 Data — (ZIP) [file pbio.1002316.s003.zip › S3_Data/FACS_Data_Fig_3A/Fig_3A_gating.pdf]

# 3C

Forward/Side Scatter

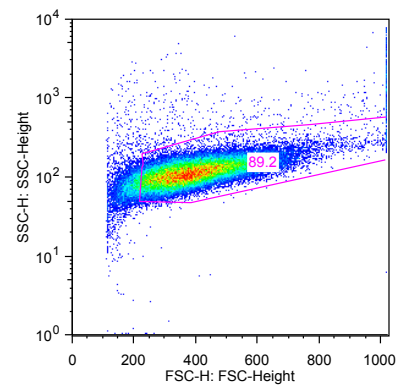

PI\_neg

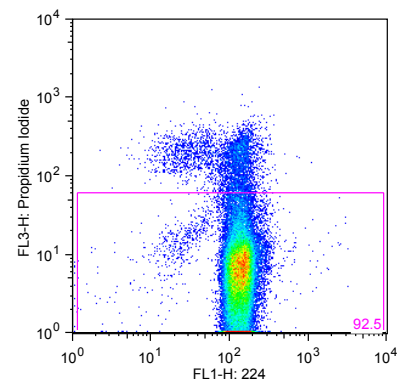

Final

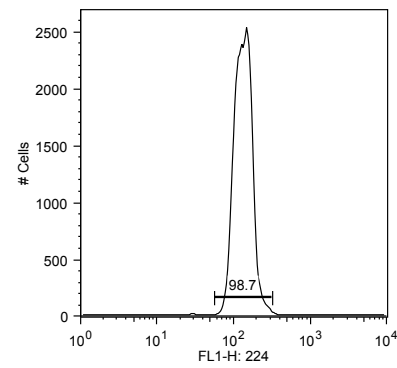

Supplement: S3 Data — (ZIP) [file pbio.1002316.s003.zip › S3_Data/FACS_Data_Fig_3C/Fig_3C_gating.pdf]

# 4

Forward/Side Scatter

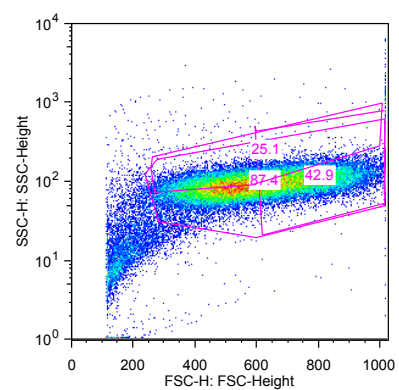

Live

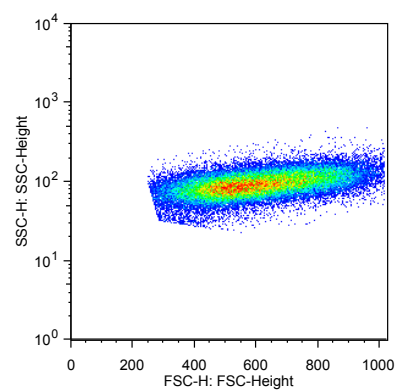

Final

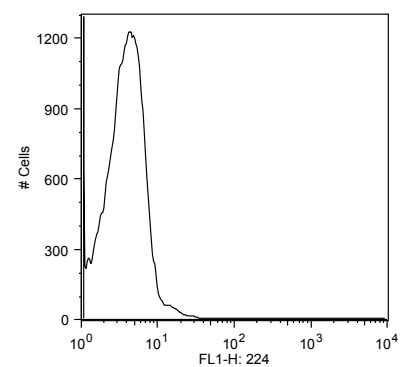

Supplement: S4 Data — (ZIP) [file pbio.1002316.s004.zip › S4_Data/FACS_Data_Fig_4/Fig_4_gating.pdf]

# 5E

Forward/Side Scatter

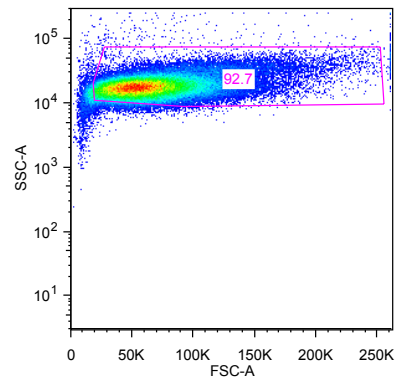

DAPI neg

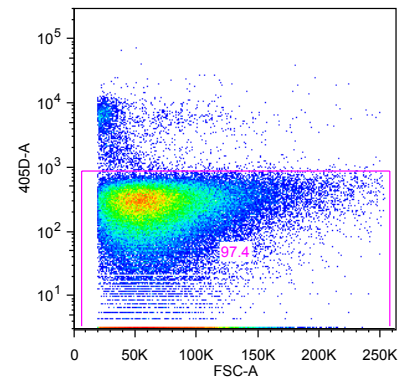

Final

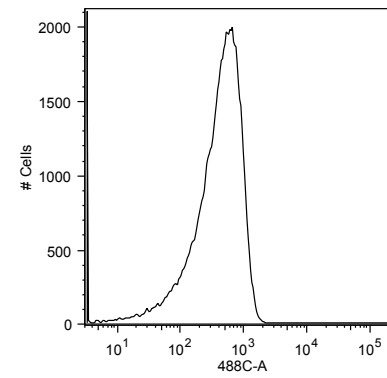

Supplement: S5 Data — (ZIP) [file pbio.1002316.s005.zip › S5_Data/FACS_Data_5E/Fig_5E_gating.pdf]

# 5F

Forward/Side Scatter

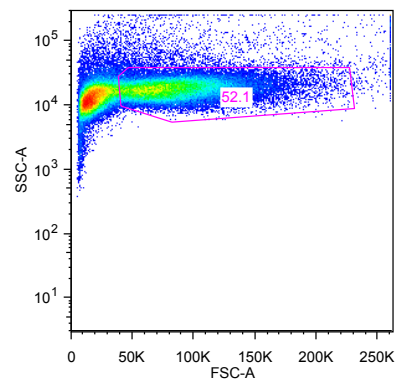

Live

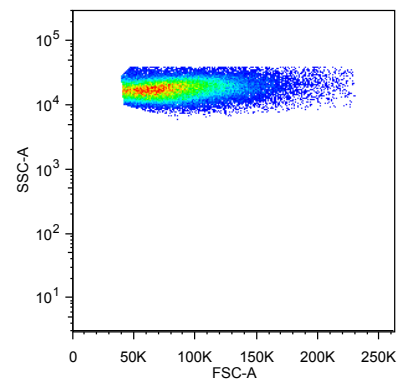

Final

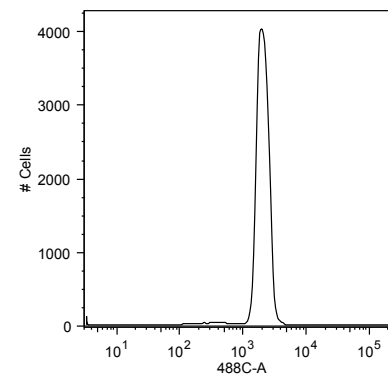

Supplement: S5 Data — (ZIP) [file pbio.1002316.s005.zip › S5_Data/FACS_Data_5F/Fig_5F_gating.pdf]

# 6B

Forward/Side Scatter

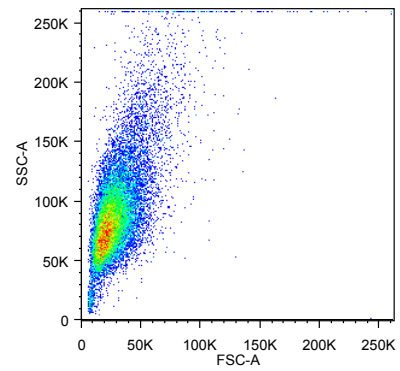

Final

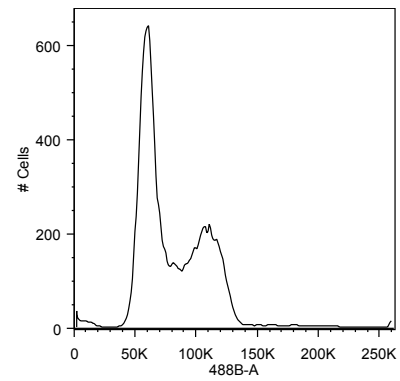

Supplement: S6 Data — (ZIP) [file pbio.1002316.s006.zip › S6_Data/FACS_Data_Fig_6B/Fig_6B_gating.pdf]

Forward/Side Scatter

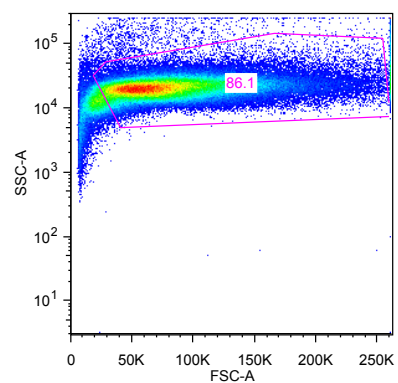

Singlets

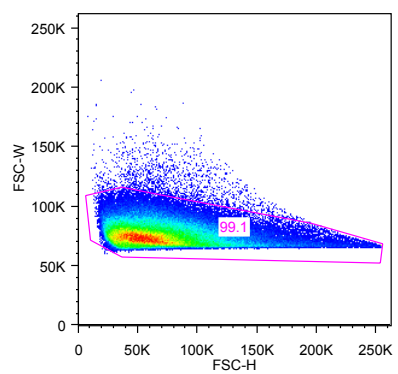

PI neg

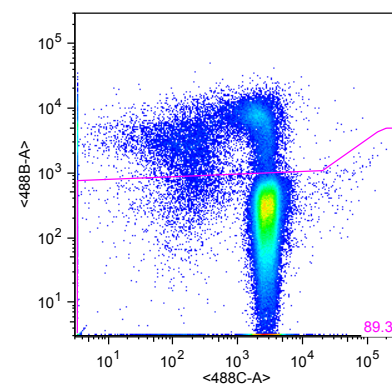

Final

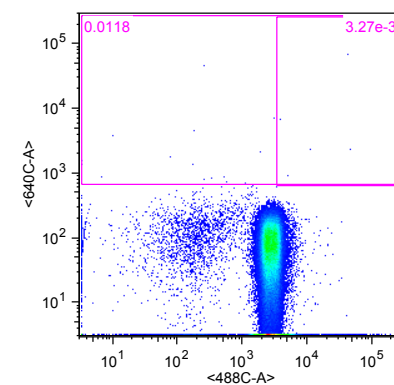

Supplement: S10 Data — (ZIP) [file pbio.1002316.s010.zip › S10_Data/FACS_Data_S4C_Fig/FACS_S4C_Fig_gating.pdf]

Live Cells

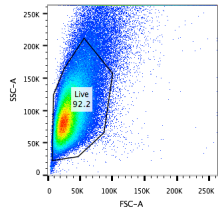

250nanomolar

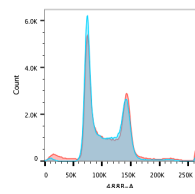

500nanomolar

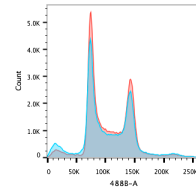

1 micromolar

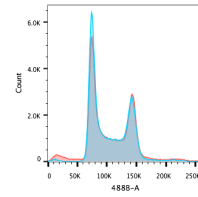

5 micromolar

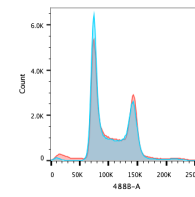

10 micromolar

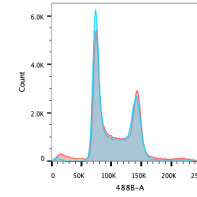

20 micromolar

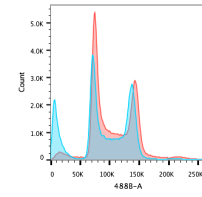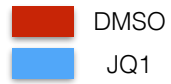

Supplement: S12 Data — (ZIP) [file pbio.1002316.s012.zip › S12_Data/JQ1_FACS_data/JQ1_cell_cycle.pdf]

# S8C

Forward/Side Scatter

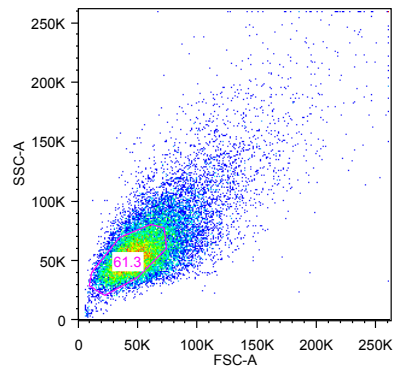

Singlets

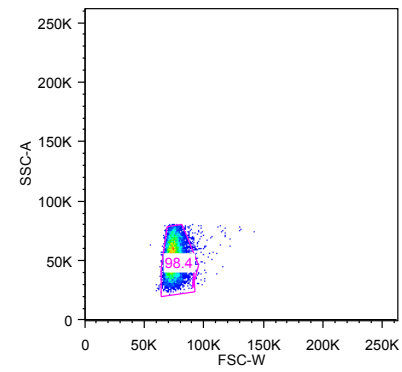

Final

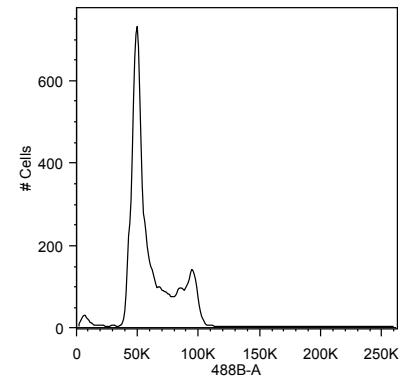

Supplement: S13 Data — (ZIP) [file pbio.1002316.s013.zip › S13_Data/S8C/S8C_Fig_gating.pdf]
