# Supplementary material for: Bromodomain Proteins Contribute to Maintenance of Bloodstream Form Stage Identity in the African Trypanosome
Source: PLoS Biol. 2015 Dec 8;13(12):e1002316. doi: 10.1371/journal.pbio.1002316 (PMC4672894; doi:10.1371/journal.pbio.1002316)
Supplement: S11 Table — (DOCX) [file pbio.1002316.s048.docx]

**Table S11. Data collection and refinement statistics.**

| **Data collection** |  |
| --- | --- |
| Synchrotron | NSLS^a^ |
| Beamline | X29 |
| Space group | P2_1_2_1_2_1_ |
| Cell dimensions |  |
| a, b, c (Å)  α, β, γ (°) | a=55.9, b=62.1, c=62.1  α=β=γ=90 |
| Wavelength (Å) | 0.97950 |
| Resolution (Å)^b^  No. of unique reflections | 50.0-1.25 (1.30-1.25)  59,818 (5,680) |
| *R*_pim_ (%)^b^ | 0.022 (39.0) |
| *<I* / σ*I>*^b^ | 37.4 (2.0) |
| Completeness (%)^b^ | 99.9 (100.0) |
| Redundancy^b^ | 7.0 (6.8) |
| **Refinement** |  |
| Resolution (Å) | 50.0 – 1.25 |
| No. of reflections  Test set | 59,801  2,035 |
| *R*_work_ / *R*_free_ (%) | 16.9 / 17.9 |
| No. of atoms | 2,113 |
| R.m.s deviations |  |
| Bond lengths (Å) | 0.006 |
| Bond angles (°)  <B-Value>  Protein (Å^2^)  I-BET151 (Å^2^)  Solvent (Å^2^) | 1.0  23.9  13.8  35.2 |
| MolProbity Score/Percentile  Ramachandran plot^c^  Favored (%)  Allowed (%)  Disallowed (%) | 1.0/100  100  0  0 |

^a^NSLS, National Synchrotron Light Source, Brookhaven National Laboratory

^b^Highest-resolution shell is shown in parentheses.

^c^As determined by MolProbity.
